# Synthesis and Characterization of Impurities of Barnidipine Hydrochloride, an Antihypertensive Drug Substance

**DOI:** 10.3390/molecules19011344

**Published:** 2014-01-21

**Authors:** Zhi-Gang Cheng, Xu-Yong Dai, Li-Wei Li, Qiong Wan, Xiang Ma, Guang-Ya Xiang

**Affiliations:** 1 School of Pharmacy, Tongji Medical College, Huazhong University of Science and Technology, Wuhan 430030, China; E-Mail: champion88@163.com; 2 Wuhan Biocause Pharmaceutical Development Co., Ltd, Wuhan 430056, Hubei, China; E-Mail: daixuyong111@126.com; 3 College of Chemical Engineering and Pharmacy, Jingchu University of Technology, Jingmen 448000, Hubei, China; E-Mail: jmliliwei@163.com; 4 Hubei Biocause Heilen Pharmaceutical Co., Ltd, Jingmen 448000, Hubei, China; E-Mail: wanqiong@biocause.net

**Keywords:** barnidipine hydrochloride, impurities, antihypertensive agent

## Abstract

Barnidipine hydrochloride is a long term dihydropyridine calcium channel blocker used for the treatment of hypertension. During the process development of barnidipine hydrochloride, four barnidipine impurities were detected by high-performance liquid chromatography (HPLC) with an ordinary column (Agilent ZORBAX Eclipse XDB-C18, 150 mm × 4.6 mm, 5 µm). All these impurities were identified, synthesized, and subsequently characterized by their respective spectral data (MS, ^1^H-NMR, and ^13^C-NMR). The identification of these impurities should be useful for quality control in the manufacture of barnidipine.

## 1. Introduction

**Figure 1:**
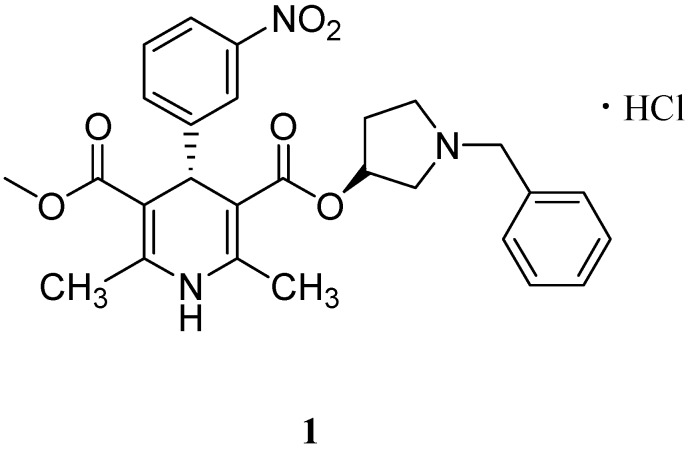
Structure of barnidipine hydrochloride (**1**).

Barnidipine hydrochloride (**1**, Figure 1), a long term dihydropyridine calcium channel blocker used for the treatment of hypertension, is chemically known as (3'*S*,4*S*)-1-benzyl-3-pyrrolidinyl-methyl-1,4-dihydro-2,6-dimethyl-4-(3-nitrophenyl)-3,5-pyridinedicarboxylate hydrochloride [1,2,3,4]. The product was originally developed by Yamanouchi Pharmaceutical (Tokyo, Japan) and is currently marketed in Japan under the trade name of Hypoca (Astellas Pharma Inc, Tokyo, Japan).

The presence of impurities in a drug substance can have a significant impact on the quality and safety of the drug product. According to the general guidelines on impurities in new drug substances recommended by the International Conference on Harmonization (ICH), the acceptable level for all impurities present should be less than 0.10% or 1.0 mg per day intake (whichever is lower) for drugs with a maximum daily dose equal to or lesser than 2 g [5]. In order to meet these requirements, the impurities present in the drug substance greater than above mentioned values must be identified and characterized. These impurities are also required in pure form to check the analytical performance characteristics, such as system suitability and relative correction factor. Hence, a comprehensive study was undertaken in our current research to identify the impurities in a sample of the barnidipine hydrochloride bulk drug substance. Their detection, synthesis, and characterization are described in this article. 

**Figure 2:**
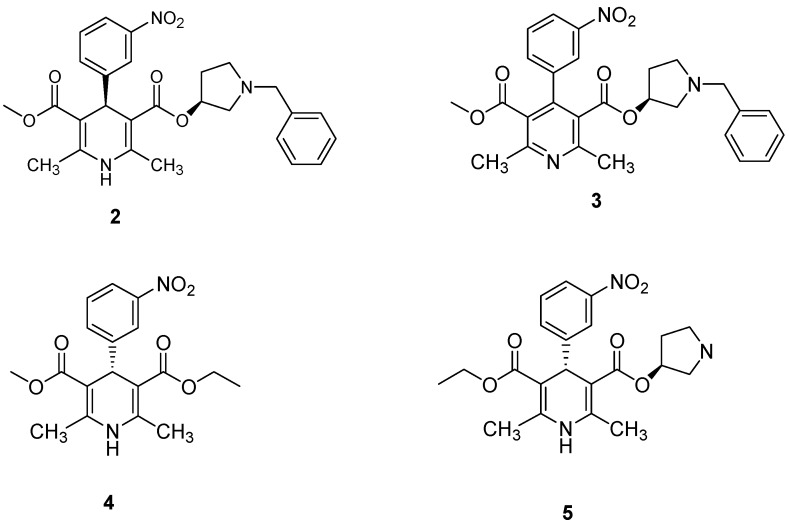
Structures of the impurities.

The four impurities identified in the barnidipine hydrochloride (**1**) manufacturing process were: (3'*S*,4*R*)-1-benzyl-3-pyrrolidinyl methyl 1,4-dihydro-2,6-dimethyl-4-(3-nitrophenyl)-3,5-pyridine dicarboxylate (**2**), 3-(*R*)-1-benzylpyrrolidin-3-yl 5-methyl 2,6-dimethyl-4-(3-nitrophenyl)pyridine-3,5-dicarboxylate (**3**), (*S*)-3-ethyl 5-methyl 2,6-dimethyl-4-(3-nitrophenyl)-1,4-dihydropyridine-3,5-dicarboxylate (**4**), and (3'*S*,4*S*)-1-benzyl-3-pyrrolidinyl ethyl 1,4-dihydro-2,6-dimethyl-4-(3-nitrophenyl)-3,5-pyridinedicarboxylate (**5**) (Figure 2). A literature survey revealed that only compounds **2** [3,6] and **3** [7,8] were previously reported, but the studies did not involve the synthesis and characterization of the compounds, so detailed synthetic processes have not been reported for the four impurities, and compounds **4** and **5** are reported here for the first time.

## 2. Results and Discussion

**Scheme 1:**
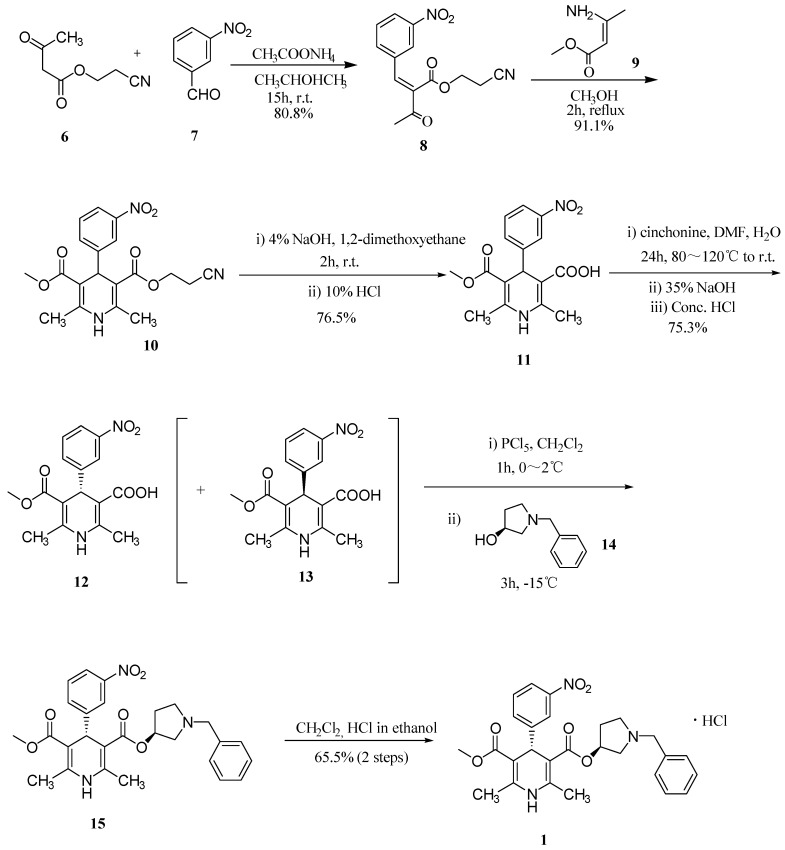
Synthesis of barnidipine hydrochloride (**1**).

In our barnidipine hydrochloride manufacturing process of (Scheme 1) [9], a key intermediate, 2-cyanoethyl 2-(3-nitrobenzylidene)-3-oxobutanoate (**8**), was obtained with a yield of 80.8% by reacting 2-cyanoethyl 3-oxobutanoate (**6**) with 3-nitrobenzaldehyde (**7**) for 15 h at room temperature. Cyclization of compound **8** with methyl 3-aminobut-2-enoate (**9**) by refluxing for 2 h afforded 3-(2-cyanoethyl) 5-methyl 2,6-dimethyl-4-(3-nitrophenyl)-1,4-dihydropyridine-3,5-dicarboxylate (**10**, yield 91.1%). Subsequent successive hydrolysis of **10** with sodium hydroxide and hydrochloric acid gave 5-(methoxycarbonyl)-2,6-dimethyl-4-(3-nitrophenyl)-1,4-dihydropyridine-3-carboxylic acid (**11**, yield 76.5%). (*R*)-5-(methoxycarbonyl)-2,6-dimethyl-4-(3-nitrophenyl)-1,4-dihydropyridine-3-carboxylic acid (**12**, optical purity 99.2%, 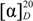 −25.9 (*c* 0.005 g/mL, acetone)) was obtained in 75.3% yield by resolution of compound **11** using cinchonine as the resolving agent. Condensation of compound 12 with (*S*)-1-benzylpyrrolidin-3-ol (**14**), and subsequent salt formation of the resulting barnidipine (**15**), provided the desired barnidipine hydrochloride (**1**, optical purity 99.8%) in 65.5% yield (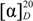 +116.5 (*c* 0.01 g/mL, MeOH)).

**Scheme 2:**
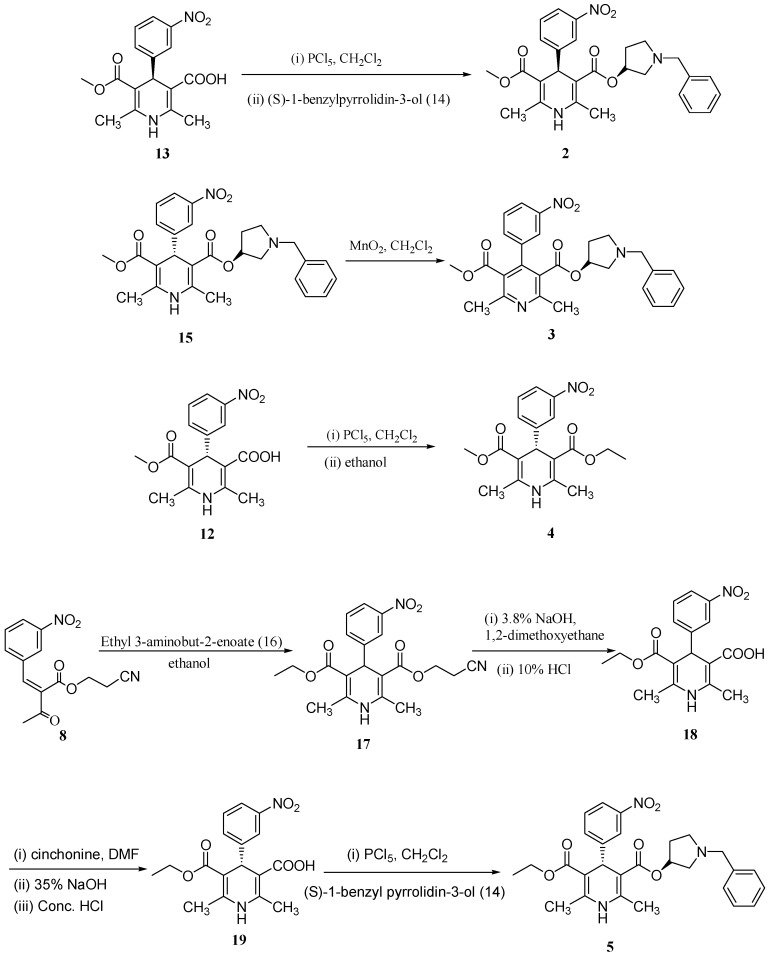
Synthesis of Barinidipine Hydrochloride Impurities.

Compound **2** is the diastereoisomer of barnidipine (**15**). The presence of (*S*)-5-(methoxycarbonyl)-2,6-dimethyl-4-(3-nitrophenyl)-1,4-dihydropyridine-3-carboxylic acid (**13**, the enantiomer of **12**) in the resolution product **12**, leads to the formation of impurity **2**. Impurity **2**, which has the same ^1^H-NMR, ^13^C-NMR, and mass spectrum as barnidipine hydrochloride, but a totally different HPLC retention time, was independently prepared using compound **13** as the starting material (Scheme 2). ^1^H-NMR, ^13^C-NMR, mass spectral (MS) data, and HPLC retention time of the prepared compound **2** were identical with those of impurity **2** separated from crude barnidipine hydrochloride (**1**). 

Compound **3** is the dehydrogenation product of barnidipine (**15**). The degradation of barnidipine explains the formation of impurity **3**. The same degradation product **3** was also reported in another study, where the barnidipine was exposed to natural or stressing irradiation, and the aromatization of the 1,4-dihydropyridine moiety in barnidine occurred [8]. This compound was prepared by reacting barnidipine (**15**) with manganese dioxide (Scheme 2). The mass spectrum of **3** showed a molecular ion at *m/z* 489.0, which is 2 amu less than that of barnidipine (**15**). The ^1^H-NMR and ^13^C-NMR spectra showed the transformation from the dihydropyridine ring to a pyridine ring. This compound was found to be identical [^1^H-NMR, ^13^C-NMR, MS and HPLC retention time (RRT 1.68, compared with **1**)] to the impurity **3** separated from crude barnidipine hydrochloride (**1**).

Compound **4** is a derivative of compound **12**. In the barnidipine hydrochloride manufacturing process, the API was recrystallized in ethanol, which explains formation of impurity **4**. This compound was prepared by reacting compound **12** with ethanol (Scheme 2). ^1^H-NMR, ^13^C-NMR, MS data, and HPLC retention time (RRT 3.14, compared with **1**) of the prepared compound **4** are identical with those of impurity **4** separated from crude barnidipine hydrochloride (**1**).

Compound **5** is the analog of barnidipine (**15**). The presence of ethyl 3-aminobut-2-enoate (**16**) in compound **9** led to the formation of impurity **5**. Compound **5** was independently prepared starting from compound **8**, following a synthetic process analogous to that of barnidipine (Scheme 2). The mass spectrum of **5** showed a molecular ion at *m/z* 505.2, which is 14 amu greater than barnidipine (**15**). The ^1^H-NMR and ^13^C-NMR spectra displayed the transformation from a methyl group to an ethyl group. This compound was found to be identical [^1^H-NMR, ^13^C-NMR, MS and HPLC retention time (RRT 1.84, compared with 1)] with the impurity **5** separated from crude barnidipine hydrochloride (**1**). Both impurities **4** and **5** might be formed due to an ester interchange reaction when ethanolic hydrochloride acid was used in the final step of the process.

## 3. Experimental

### General Information

The ^1^H-NMR spectra were recorded on a Varian Mercury plus-400 MHz Fourier transform (FT)-NMR spectrometer, and chemical shift values were reported as δ ppm relative to tetramethylsilane (TMS). The ^13^C-NMR spectra were recorded on a Varian Mercury plus-400 MHz Fourier transform (FT)–NMR spectrometer, and chemical shift values were reported on δ ppm relative to CDCl_3_ or DMSO-*d*_6_. The mass spectra were recorded on an Agilent API 2000LC-MS/MS mass spectrometer. The HPLC chromatograms were recorded on a Waters 1525 HPLC instrument. 

*(3'S,4R)-1-Benzyl-3-pyrrolidinyl methyl 1,4-dihydro-2,6-dimethyl-4-(3-nitrophenyl)-3,5-pyridine-dicarboxylate* (**2**) Phosphorus pentachloride (1.95 g, 0.02 mmol) was added slowly to a solution of (*S*)-5-(methoxycarbonyl)-2,6-dimethyl-4-(3-nitrophenyl)-1,4-dihydropyridine-3-carboxylic acid (**13**, 2.47 g, 0.0074 mol) in dichloromethane (DCM, 32.5 mL) at −20 °C, and stirred for 1 h below −15 °C. Then the mixture was cooled to −26 °C, and a solution of (*S*)-1-benzylpyrrolidin-3-ol (**14**, 1.35 g, 0.0076 mol) and dichloromethane (11.25 mL) were added to above mixture, and stirred for 2 h. The reaction mixture was washed with 5% sodium carbonate solution (150 mL) and water (150 mL). The organic layer was dried over anhydrous magnesium sulfate and concentrated under reduced pressure at less than 35 °C. The residue was purified by flash column chromatography on silica gel, eluting with ethyl acetate/petroleum ether, 3:2, to yield **2** as a light yellow solid (1.5g, 41%). HPLC purity 92.9%. ^1^H-NMR (CDCl_3_) δ: 7.27–8.10 (m, 9H), 5.90 (s, 1H), 5.10–5.14 (m, 1H), 5.08 (s, 1H), 3.64 (s, 3H), 3.51–3.58 (m, 2H), 2.74–2.79 (m, 1H), 2.66–2.70 (m, 1H), 2.48–2.51 (m, 1H), 2.35 (s, 6H), 2.10–2.28 (m, 2H), 1.85-1.86 (m, 1H); ^13^C-NMR (CDCl_3_) δ: 167.5, 166.8, 149.8, 148.4, 144.9, 144.8, 138.8, 134.5, 128.7, 128.6, 128.2, 127.0, 122.9, 121.3, 103.3, 73.8, 60.1, 59.8, 52.6, 51.1, 39.9, 29.7, 19.6. MS (ESI−) *m/z*: 490.1 (M−H)^−^.

*3-(R)-1-Benzylpyrrolidin-3-yl 5-methyl 2,6-dimethyl-4-(3-nitrophenyl) pyridine-3,5-dicarboxylate* (**3**) Manganese dioxide (3.5 g, 0.04 mol) was added to a solution of barnidipine (**15**, 2.0 g, 0.004 mol) in dichloromethane (150 mL), exposed to UV light, and stirred for 30 days at room temperature. The reaction mixture was filtered and concentrated. The residue was purified by flash column chromatography on silica gel, eluting with dichloromethane/ethanol, 40:1, followed by ethyl acetate/petroleum ether, 3:2, to afford **3** as a light yellow solid (0.6 g, 31%). HPLC purity 94.6%. ^1^H-NMR (CDCl_3_) δ: 7.13–8.10 (m, 9H), 4.96–4.99 (m, 1H), 3.34–3.47 (m, 5H), 2.46–2.51 (m, 8H), 2.21–2.27 (m, 1H), 2.11–2.14 (m, 1H), 1.92–2.03 (m, 1H), 1.37–1.43 (m, 1H). ^13^C-NMR (CDCl_3_) δ: 167.4, 166.7, 156.1, 156.0, 147.6, 143.4, 138.1, 137.8, 134.1, 128.1, 128.5, 128.0, 126.1, 126.1, 123.2, 123.0, 75.4, 59.6, 58.9, 52.2, 52.1, 31.1, 22.9. MS (ESI+) *m/z*: 490.0 (M+H)^+^.

*(S)-3-Ethyl 5-methyl 2,6-dimethyl-4-(3-nitrophenyl)-1,4-dihydropyridine-3,5-dicarboxylate* (**4**). Phosphorus pentachloride (1.66 g, 0.017 mol) was added to the solution of compound **12** (2.1 g, 0.006 mol) in dichloromethane (28 mL) below −20 °C, and stirred for 1 h. The reaction mixture was cooled to −30 °C, and was added ethanol (10 mL, 0.17 mol), and stirred for 4 h. The reaction mixture was poured into saturated sodium carbonate solution (200 mL), and extracted with dichloromethane (200 mL). The organic phase was washed with water (300 mL), dried over anhydrous magnesium sulfate and concentrated. The residue was purified by flash column chromatography on silica gel, eluting with ethyl acetate/petroleum ether, 3:2, to yield **4** as a light yellow solid (1.2 g, 55%). HPLC purity 99.7%. ^1^H-NMR (DMSO-d6) δ: 9.04 (s, 1H), 7.52-8.01 (m, 4H), 4.99 (s, 1H), 3.98–4.03 (m, 2H), 3.55 (s, 3H), 2.30 (s, 6H), 1.12–1.16 (m, 3H). ^13^C-NMR (DMSO-*d*_6_) δ: 167.7, 167.1, 150.7, 148.2, 147.2, 147.0, 134.7, 130.3, 122.3, 121.8, 101.7, 101.4, 59.9, 51.4, 41.0, 18.9 (2C), 14.7. MS (ESI−) *m/z*: 359.1 (M−H)^−^.

*3-(2-Cyanoethyl) 5-ethyl 2,6-dimethyl-4-(3-nitrophenyl)-1,4-dihydro-pyridine-3,5-dicarboxylate* (**17**) Ethyl 3-aminobut-2-enoate (**16**, 37.3 g, 0.29 mol) was added to the solution of compound **8** (85 g, 0.295 mol) in ethanol (216 mL), heated to reflux for 2 h. The reaction mixture was concentrated at 60 °C to about 100 mL, and cooled to 0–5 °C. The precipitate was collected by vacuum filtration and dried at 60 °C for 10 h to provide 17 (45.4 g, 39%). ^1^H-NMR (DMSO-*d*_6_) δ: 9.09 (s, 1H), 7.48–7.99 (m, 4H), 4.97(s, 1H), 4.09–4.13 (m, 2H), 3.93–4.0 (m, 2H), 2.77–2.83 (m, 2H), 2.27–2.30 (d, 6H), 1.10–1.15 (m, 3H). ^13^C-NMR (DMSO-*d*_6_) δ: 167.1, 166.6, 150.7(2C), 148.3, 146.8, 134.9, 130.3, 122.6, 121.8, 119.2, 102.2, 100.9, 59.9, 59.2, 41.0, 19.1, 18.8, 18.0, 14.7. MS (ESI−) *m/z*: 398.2 (M−H)^−^.

*5-(Ethoxycarbonyl)-2,6-dimethyl-4-(3-nitrophenyl)-1,4-dihydropyridine-3-carboxylic acid* (**18**) Compound **17** (45 g, 0.11 mol) was added to the mixture of 3.8% sodium hydroxide solution (350 g) and 1,2-dimethoxyethane (170 mL) at 30 °C, stirred for 2 h. The reaction mixture was diluted with water (115 mL), and extracted with dichloromethane (230 mL). The aqueous phase was acidized to pH 3.0 by 10% hydrochloric acid. The precipitate was filtered and washed with water, and dried at 90 °C for 2 h to afford 18 (31.8 g, 83.5%). ^1^H-NMR (DMSO-*d*_6_) δ:11.82 (s, 1H), 8.91 (s, 1H), 7.50–7.98 (m, 4H), 4.98 (s, 1H), 3.97–4.01 (m, 2H), 2.28 (s, 6H), 1.11–1.15 (m, 3H). ^13^C-NMR (DMSO-*d_6_*) δ: 169.1, 167.2, 150.9, 148.1, 147.2, 146.4, 134.8, 130.2, 122.4, 121.7, 102.3, 101.3, 59.8, 41.0, 18.9(2C), 14.7. MS (ESI−) *m/z*: 345.1 (M−H)^−^.

*(R)-5-(Ethoxycarbonyl)-2,6-dimethyl-4-(3-nitrophenyl)-1,4-dihydropyridine-3-carboxylic acid* (**19**) Cinchonine (29.4 g, 0.10 mol) was added to the solution of compound **18** (31.0 g, 0.09 mol) in *N,N*-dimethylformamide (5.7 mL), and heated 80 °C to get a clear solution. Water (38.4 mL) was added, and heated to 120 °C. The solution was cooled slowly to 20 °C and stirred for 12 h. The precipitate was filtered and washed with *N*,*N*-dimethylformamide/water (3:2) solution (12 mL). The filter mass was dissolved in 35% sodium hydroxide solution (92 mL), extracted with dichloromethane (80 mL). The aqueous phase was acidized to pH 2.0 with concentrated hydrochloric acid. The precipitate was collected and washed with water, and dried at 100 °C for 10 h to provide **19** (10.4 g, 33%). ^1^H-NMR (DMSO-*d_6_*) δ: 11.82 (s, 1H), 8.91 (s, 1H), 7.50–7.99 (m, 4H), 4.97 (s, 1H), 3.96–4.00 (m, 2H), 2.27 (s, 6H), 1.10–1.15 (m, 3H); ^13^C-NMR (DMSO-*d_6_*) δ: 169.1, 167.2, 150.9, 148.1, 147.2, 146.4, 134.8, 130.2, 122.4, 121.7, 102.3, 101.3, 59.8, 41.0, 18.9 (2C), 14.8. MS (ESI−) *m/z*: 345.1 (M−H)^−^.

*(3'S,4S)-1-Benzyl-3-pyrrolidinyl ethyl 1,4-dihydro-2,6-dimethyl-4-(3-nitrophenyl)-3,5-pyridine-dicarboxylate* (**5**). Phosphorus pentachloride (3.5 g, 0.036 mol) was added to the solution of compound **19** (4.8 g, 0.014 mol) in dichloromethane (70 mL) below 0 °C, and stirred for 1 h. The reaction mixture was cooled to −20 °C, and was added the solution of (*S*)-1-benzylpyrrolidin-3-ol (**14**, 2.5 g, 0.014 mol) in dichloromethane (70 mL), and stirred for 2 h. The reaction mixture was poured into saturated sodium carbonate solution (275 mL), and extracted with dichloromethane (275 mL). The organic phase was washed with water (550 mL), dried over anhydrous magnesium sulfate and concentrated. The residue was purified by flash column chromatography on silica gel, eluting with ethyl acetate/petroleum ether, 3:2, to yield **5** as a light yellow solid (2.7 g, 38%). HPLC purity 98.2%. ^1^H-NMR (CDCl_3_) δ: 7.23–8.13 (m, 9H), 6.58 (br, 1H), 5.07–5.11 (m, 2H), 4.05–4.10 (m, 2H), 3.60–3.66 (m, 2H), 2.80–2.83 (m, 1H), 2.61–2.63 (m, 2H), 2.42–2.44 (m, 1H), 2.30 (s, 6H), 2.06–2.17 (m, 1H), 1.52–1.71 (m, 1H), 1.19–1.25 (m, 3H). ^13^C-NMR (CDCl_3_) δ: 167.1, 166.8, 150.0, 148.0, 145.2, 144.9, 138.7, 134.5, 128.5, 128.4, 128.1, 126.8, 122.9, 121.1, 103.0, 102.8, 77.0, 73.6, 60.0, 59.8, 52.4, 39.9, 31.7, 19.1, 14.1. MS (ESI−) *m/z*: 504.2 (M−H)^−^.

## 4. Conclusions

Information on different potential impurities and their synthetic routes is very important for better understanding of the impurity formation pathways of the antihypertensive drug barnidipine hydrochloride. All the impurities were identified, synthesized, and subsequently characterized by their respective spectral data. Keeping in mind the regulatory importance of barnidipine hydrochloride impurities, our efforts to synthesize and characterize them effectively should prove to be valuable.

## Supplementary Materials

Supplementary materials can be accessed at: http://www.mdpi.com/1420-3049/19/1/1344/s1.

## References

[1] Huh W.S., Kim Y.S., Han J.S., Kim S.G., Kim S.B., Park J.S., Yamamoto M. (2000). Antihypertensive efficacy and tolerability of barnidipine hydrochloride in patients with renal parenchymal hypertensio. Curr. Ther. Res..

[2] Imai Y., Abe K., Nishiyama A., Sekino M., Yoshinaga K. (1997). Evaluation of the antihypertensive effect of barnidipine, a dihydropyridine calcium entry blocker, as determined by the ambulatory blood pressure level averaged for 24 h, daytime, and nigttime. Am. J. Hypertens..

[3] Tamazawa K., Arima H., Kojima T., Isomura Y., Okada M., Fujita S., Furuya T., Takenaka T., Inagaki O., Terai M. (1986). Stereoselectivity of a potent calcium antagonist, 1-benzyl-3-pyrrolidinyl methyl 2,6-dimethyl-4-(*m*-nitrophenyl)-1,4-dihydropyridine-3,5-dicarboxylate. J. Med. Chem..

[4] Kojima T., Takenaka T. 1,4-Dihydropyridine-3,5-dicarboxylic Acid Ester Derivatives. U.S. Patent.

[5] ICH Harmonised Tripartite Guideline: Impurities in new drug substances Q3A(R2). Proceedings of the International Conference on Harmonization of Technical Requirements for Registration of Pharmaceuticals for Human Use.

[6] Inagaki O., Asano M., Takenaka T. (1999). *In vitro* and *in vivo* vasodilatory activity of barnidipine and its enantiomers. Biol. Pharm. Bull..

[7] Pawula M., Watson D., Teramura T., Watanabe T., Higuchi S., Cheng K.N. (1998). Sensitive and specific liquid chromatographic-tandem mass spectrometric assay for barnidipine in human plasma. J. Chrom. B Biomed. Sci. Appl..

[8] Ioele G., Oliverio F., Andreu I., de Luca M., Miranda M.A., Ragno G. (2010). Different photodegradation behavior of barnidipine under natural and forced irradiation. J. Photochem. Photobiol. A Chem..

[9] Li L., Cheng Z., Li X., Gao J., Su C., Ma X. (2010). Synthesis Process of Barnidipine Hydrochloride. CN Patent.

