# Supplementary material for: Synthesis and Characterization of Impurities of Barnidipine Hydrochloride, an Antihypertensive Drug Substance
Source: Molecules. 2014 Jan 21;19(1):1344–52. doi: 10.3390/molecules19011344 (PMC6270734; doi:10.3390/molecules19011344)

Supporting Information

Supporting Information 1:

HPLC Analytical Method
Chromatographic condition
Column:
-size: l = 0.15 m, Φ = 4.6 mm
-stationary phase: octadecylsilyl silica gel for chromatography (5 μm)
Mobile phase: mix 50 volume of methanol and 50 volume of 0.03 mol/L monopotassium phosphate buffer (adding 2 mL of triethylamine to 1000 mL of 0.03 mol/L mono potassium solution, ajust pH to 4.0 by phosphoric acid).
Injection volumn: 10 μL
Flow rate: 1.2 mL/min
Detection: spectrophotometer at 238 nm
Test solution: with the aid of ultrasound, dissolve 25 mg of the substance to be examined in 40 mL of mobile phase and dilute to 50.0 mL with the same solvent.

Supporting information 2: Chromatograms and spectrograms

Figure S1. HPLC Chromatogram of Impurity **2**.

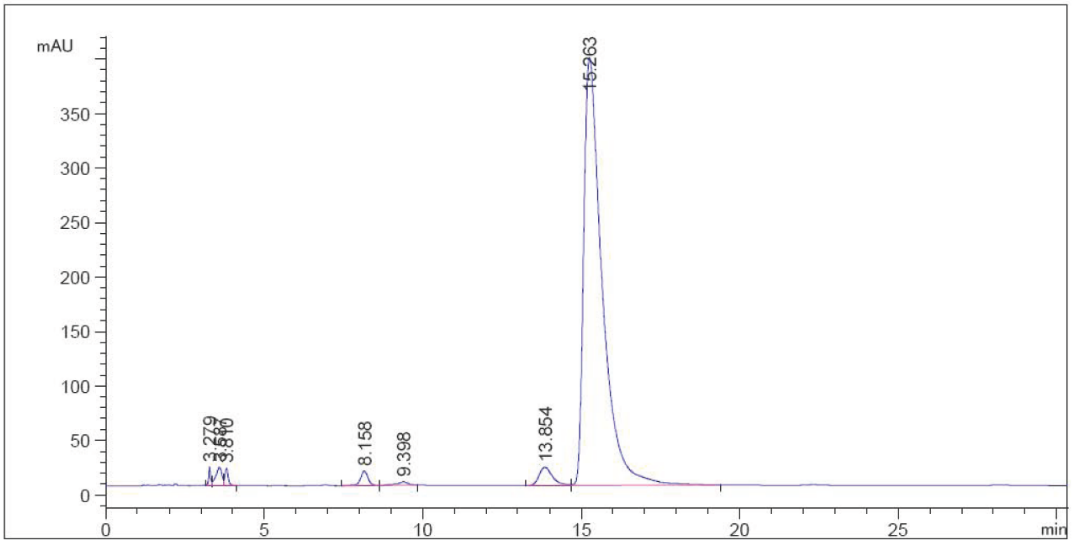

Area Percent Report

Sorted By : Signal
Multiplier : 1.0000
Dilution : 1.0000
Use Multiplier & Dilution Factor with ISTDs

Signal 1: VWD1 A, Wavelength=238 nm

Peak #	RetTime [min]	Type	Width [min]	Area mAU *s	Height [mAU]	Area %
1	3.279	BV	0.0604	71.04477	17.00593	0.4145
2	3.587	VV	0.1888	220.21754	16.85555	1.2850
3	3.810	VB	0.1063	114.43090	16.08986	0.6677
4	8.158	BB	0.2565	225.03416	13.34277	1.3131
5	9.398	BB	0.3573	77.53874	3.08524	0.4524
6	13.854	BV	0.4416	503.77908	17.01027	2.9395
7	15.263	VB	0.5940	1.59261e4	392.69418	92.9278
Totals :				1.71381e4	476.08381	

*** End of Report ***

Figure S2. MS spectrogram of Impurity **2**.

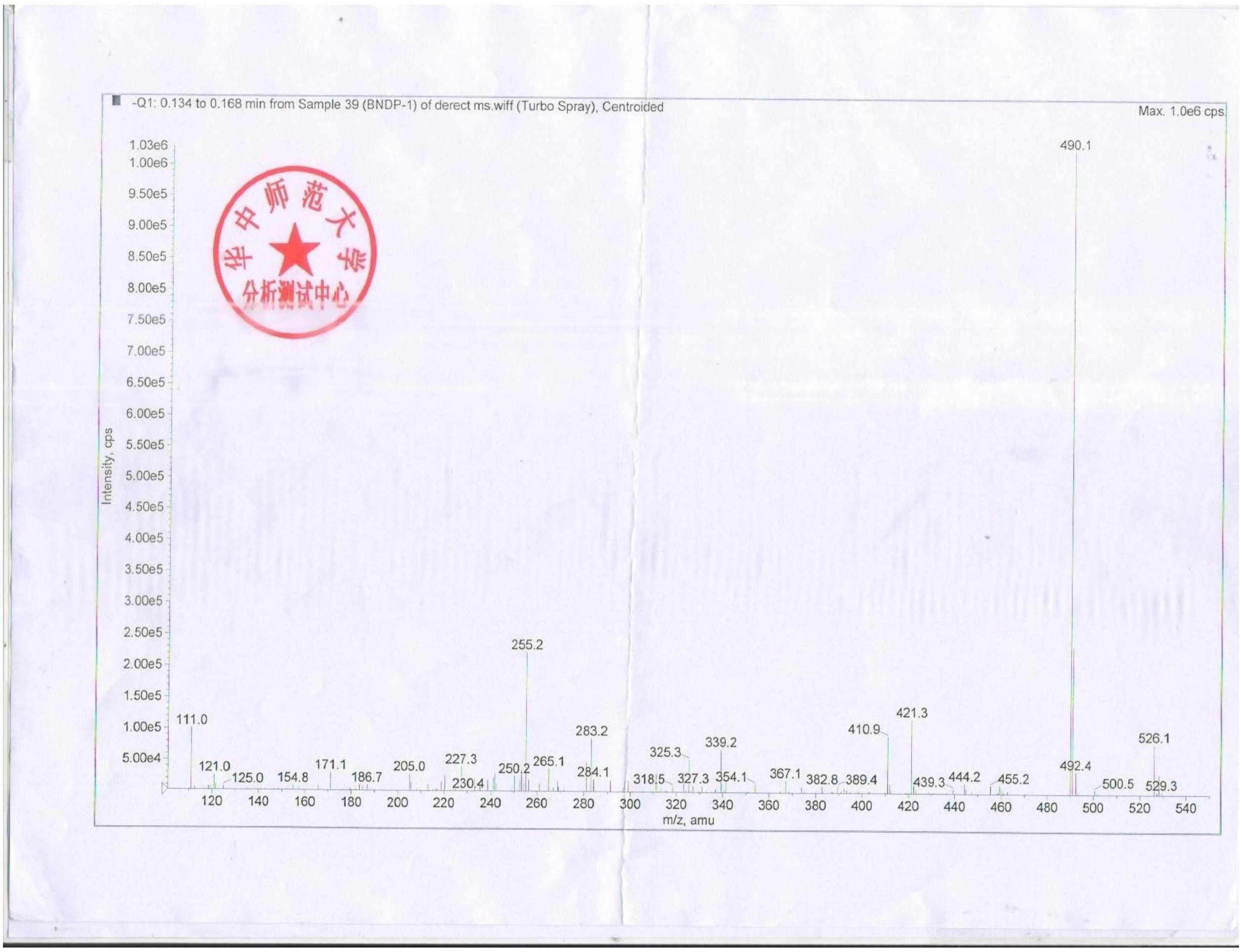

Figure S3. ^{1}H-NMR spectrogram of Impurity **2** in $CDCl_3$.

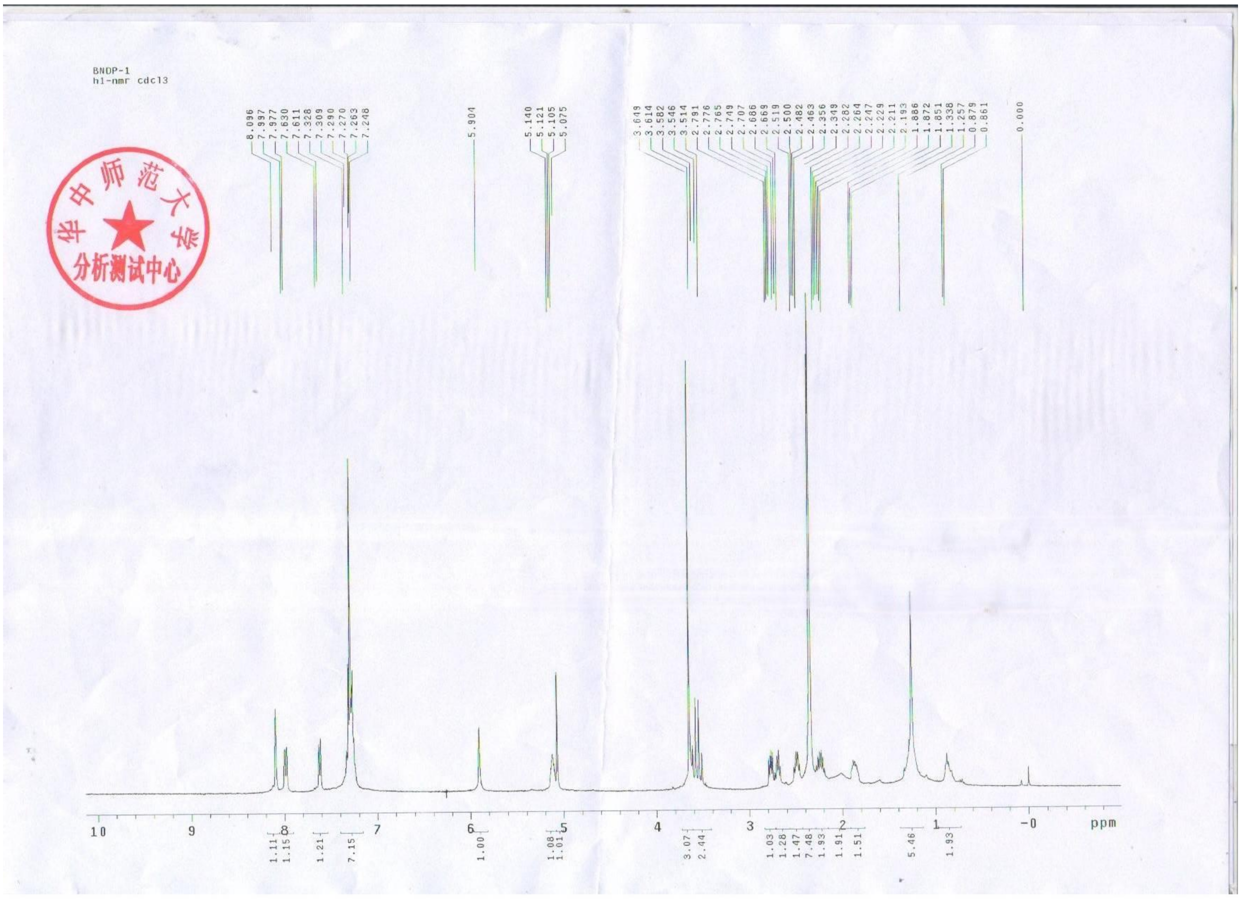

Figure S4. ^{13}C-NMR spectrogram of Impurity **2** in $CDCl_3$.

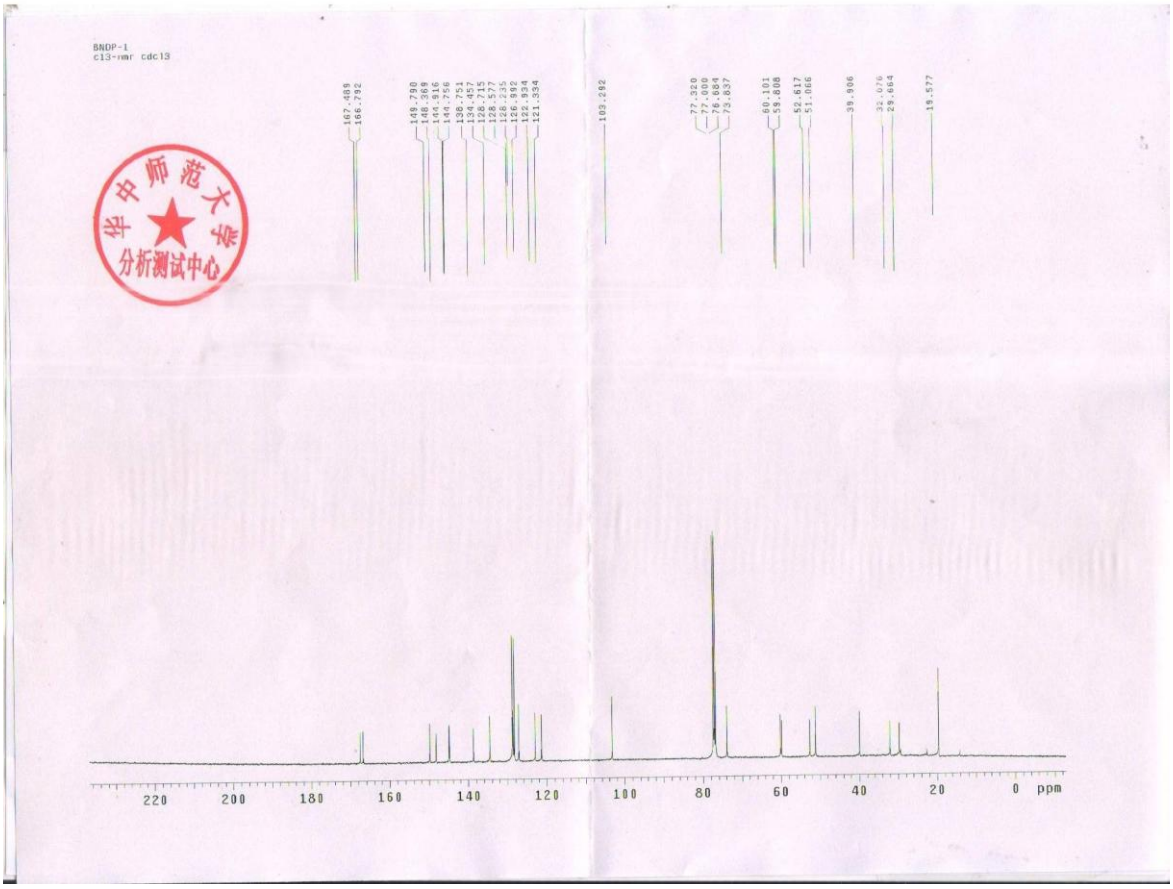

Figure S5. HPLC Chromatogram of Impurity **3**.

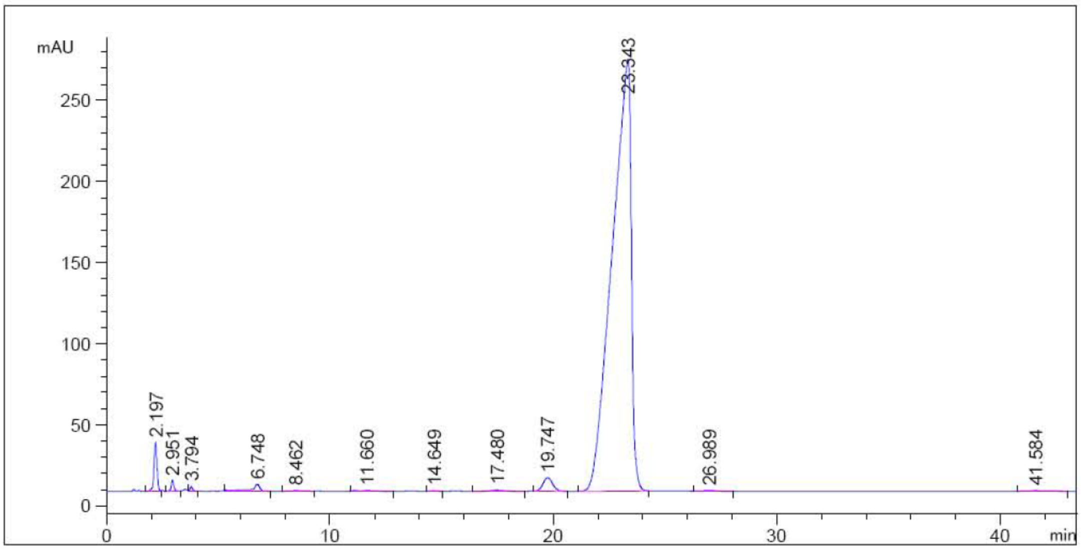

===

Area Percent Report

===

Sorted By : Signal
Multiplier : 1.0000
Dilution : 1.0000
Use Multiplier & Dilution Factor with ISTDs

Signal 1: VWD1 A, Wavelength=238 nm

Peak #	RetTime [min]	Type	Width [min]	Area mAU *s	Height [mAU]	Area %
1	2.197	BV	0.1488	289.90604	30.10201	1.7338
2	2.951	BB	0.1204	55.88548	6.81855	0.3342
3	3.794	VB	0.1239	22.99636	2.78789	0.1375
4	6.748	MM	0.4624	119.61582	4.31179	0.7154
5	8.462	MM	0.5695	18.56132	5.43189e-1	0.1110
6	11.660	MM	1.0091	39.07751	6.45429e-1	0.2337
7	14.649	MM	0.3939	10.86778	4.59875e-1	0.0650
8	17.480	MM	0.6898	34.83944	8.41755e-1	0.2084
9	19.747	BB	0.4542	254.21104	8.38398	1.5204
10	23.343	MM	0.9893	1.58171e4	266.45694	94.5969
11	26.989	MM	0.6619	23.79769	5.99192e-1	0.1423
12	41.584	MM	1.1595	33.66652	4.83932e-1	0.2013
Totals :				1.67205e4	322.43453	

===

*** End of Report ***

Figure S6. MS spectrogram of Impurity **3**.

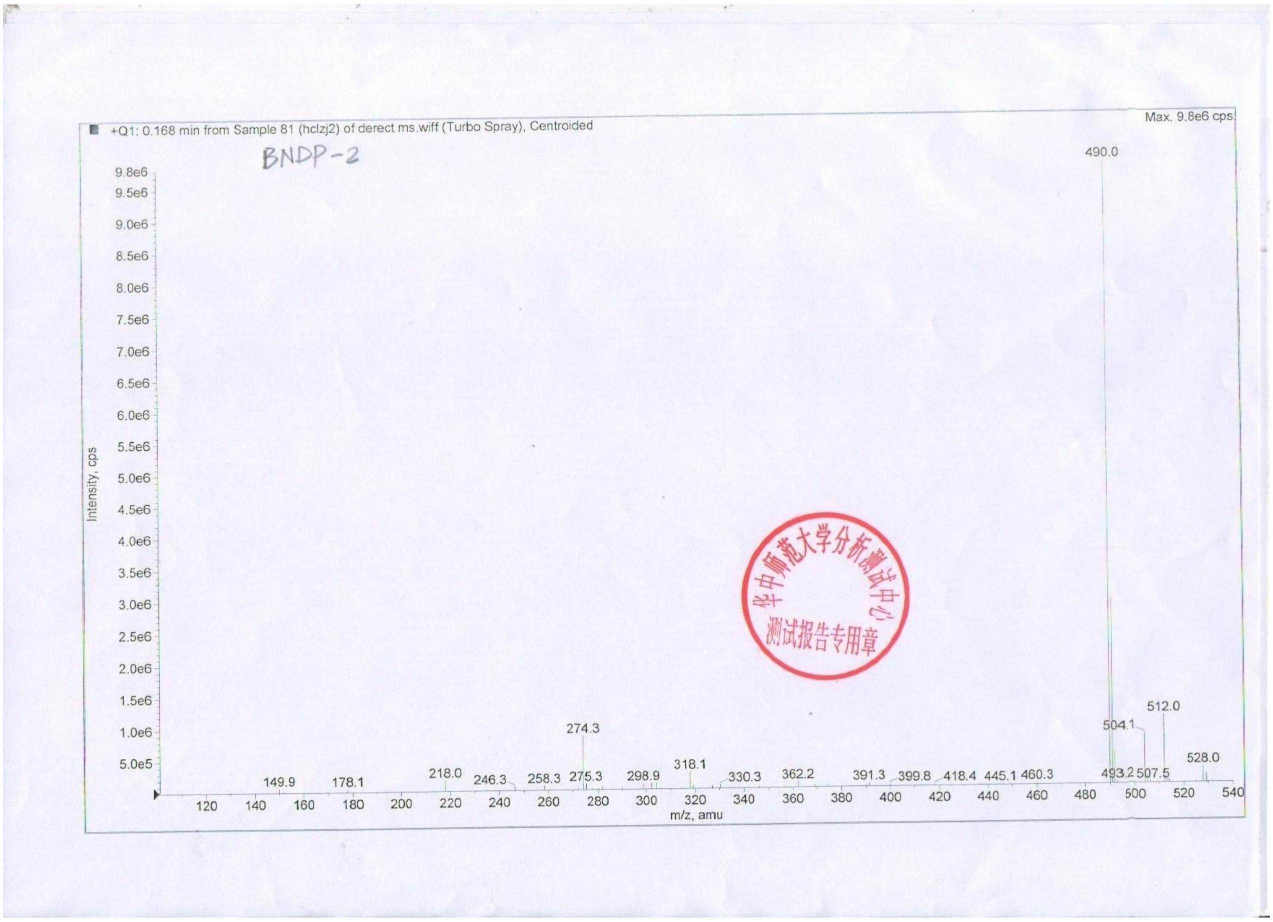

Figure S7. ^{1}H-NMR spectrogram of Impurity **3** in $CDCl_3$.

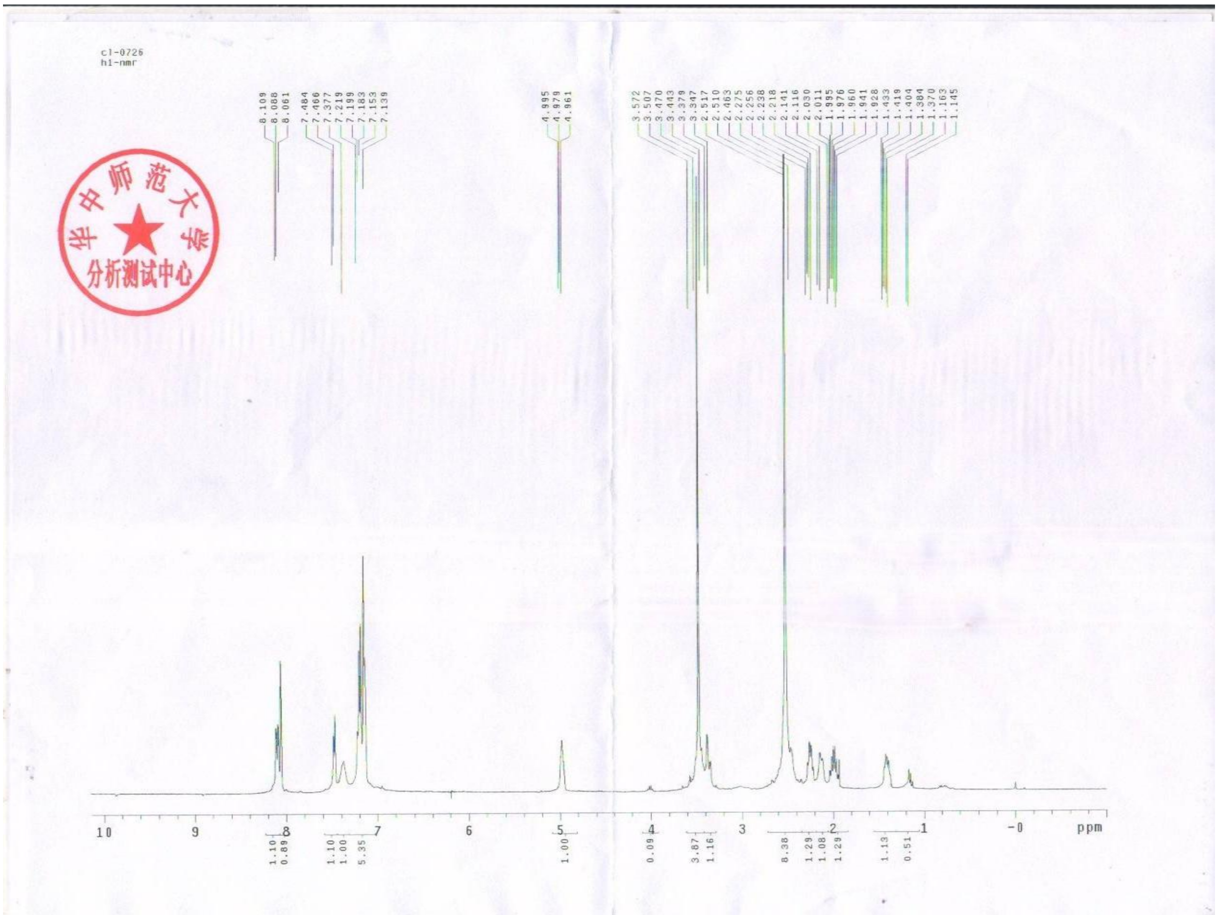

Figure S8. ^{13}C-NMR spectrogram of Impurity **3** in $CDCl_3$.

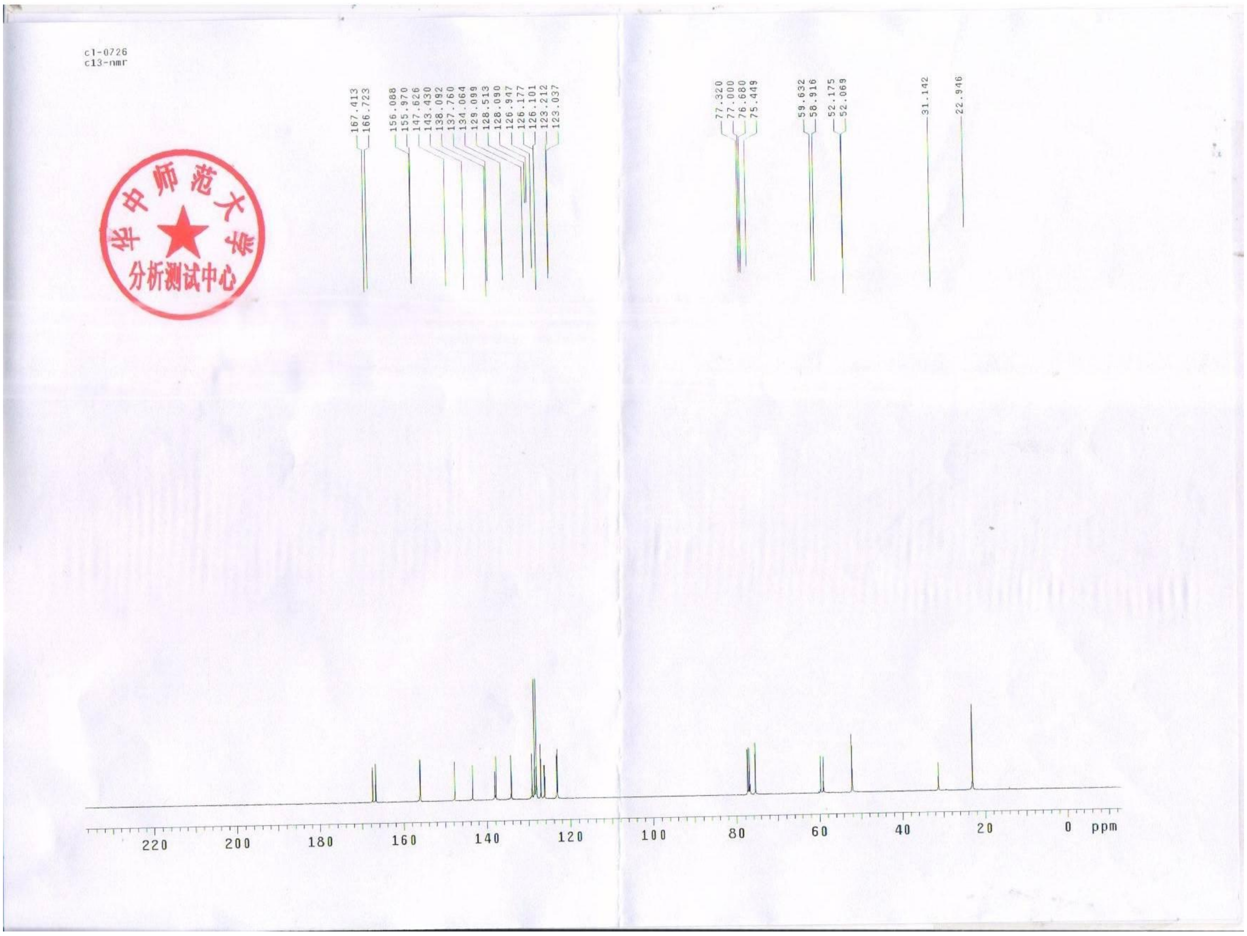

Figure S9. HPLC Chromatogram of Impurity **4**.

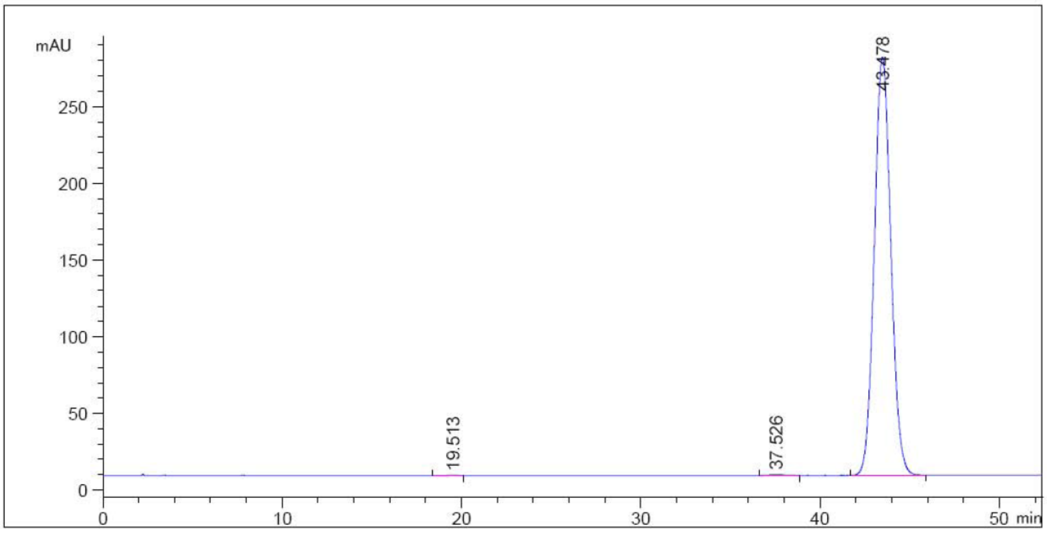

Area Percent Report

Sorted By : Signal
Multiplier : 1.0000
Dilution : 1.0000
Use Multiplier & Dilution Factor with ISTDs

Signal 1: VWD1 A, Wavelength=238 nm

Peak #	RetTime [min]	Type	Width [min]	Area mAU *s	Height [mAU]	Area %
1	19.513	MM	0.4490	9.78214	3.63075e-1	0.0557
2	37.526	MM	1.0145	41.08688	6.74983e-1	0.2338
3	43.478	BB	0.9987	1.75203e4	272.64084	99.7105
Totals :				1.75711e4	273.67890	

*** End of Report ***

Figure S10. MS spectrogram of Impurity **4**.

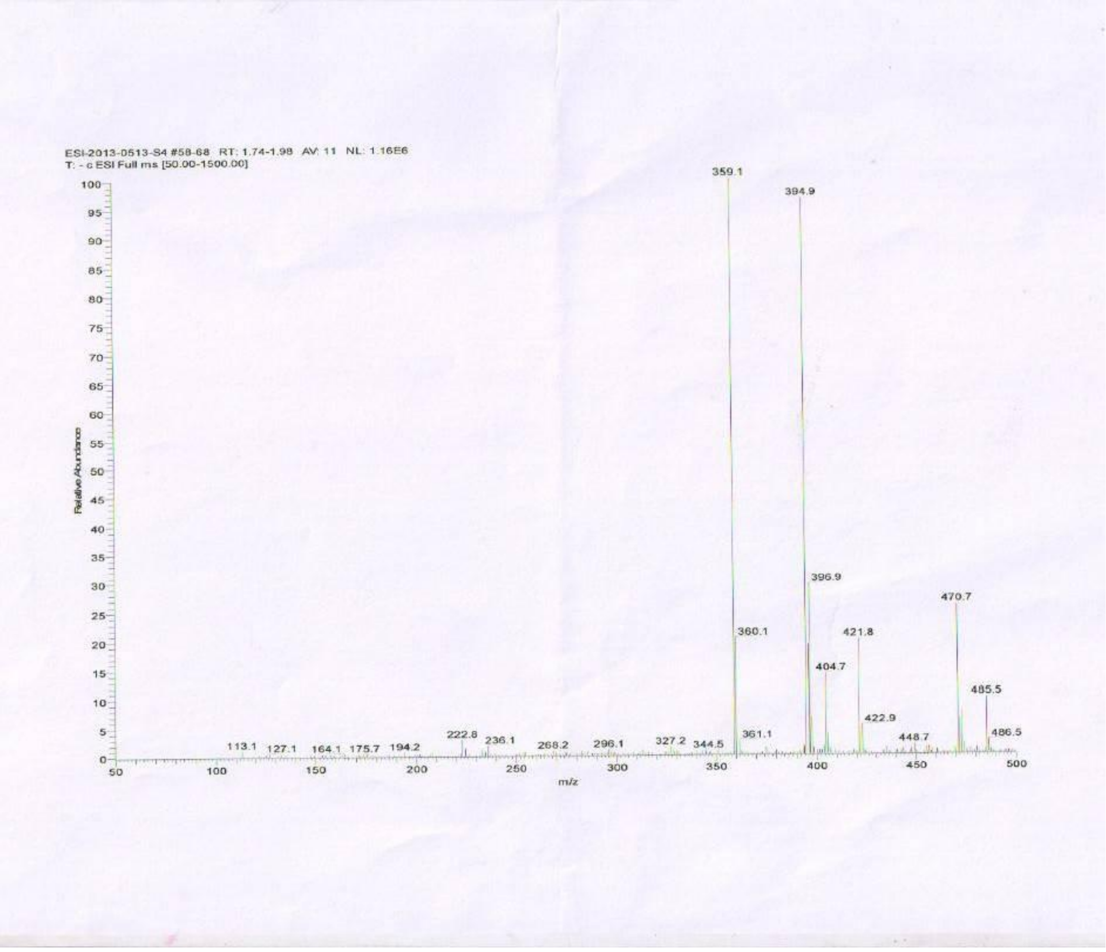

Figure S11. ^{1}H-NMR spectrogram of Impurity **4** in DMSO-d_6.

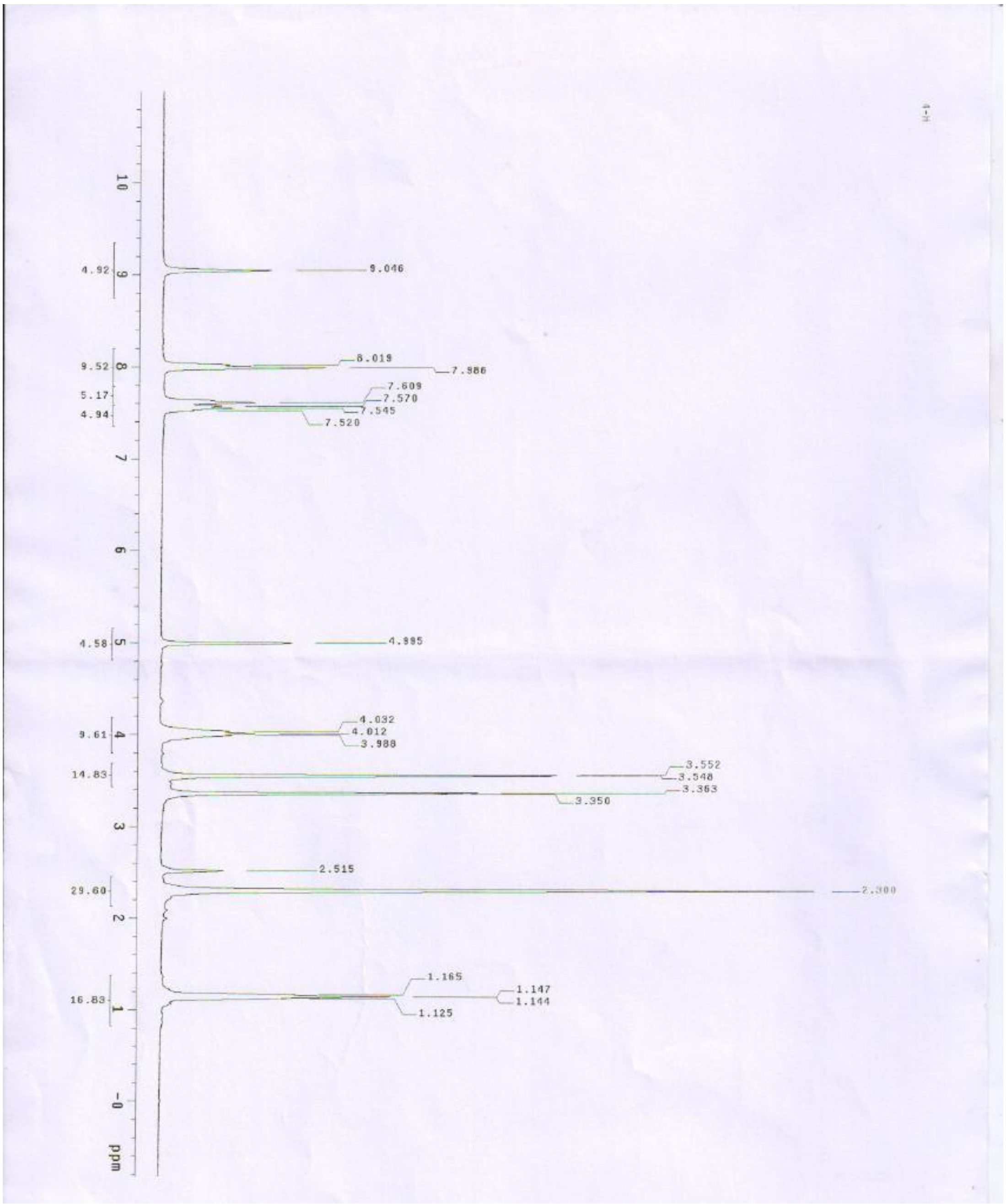

Figure S12. ^{13}C-NMR spectrogram of Impurity **4** in DMSO-d_6.

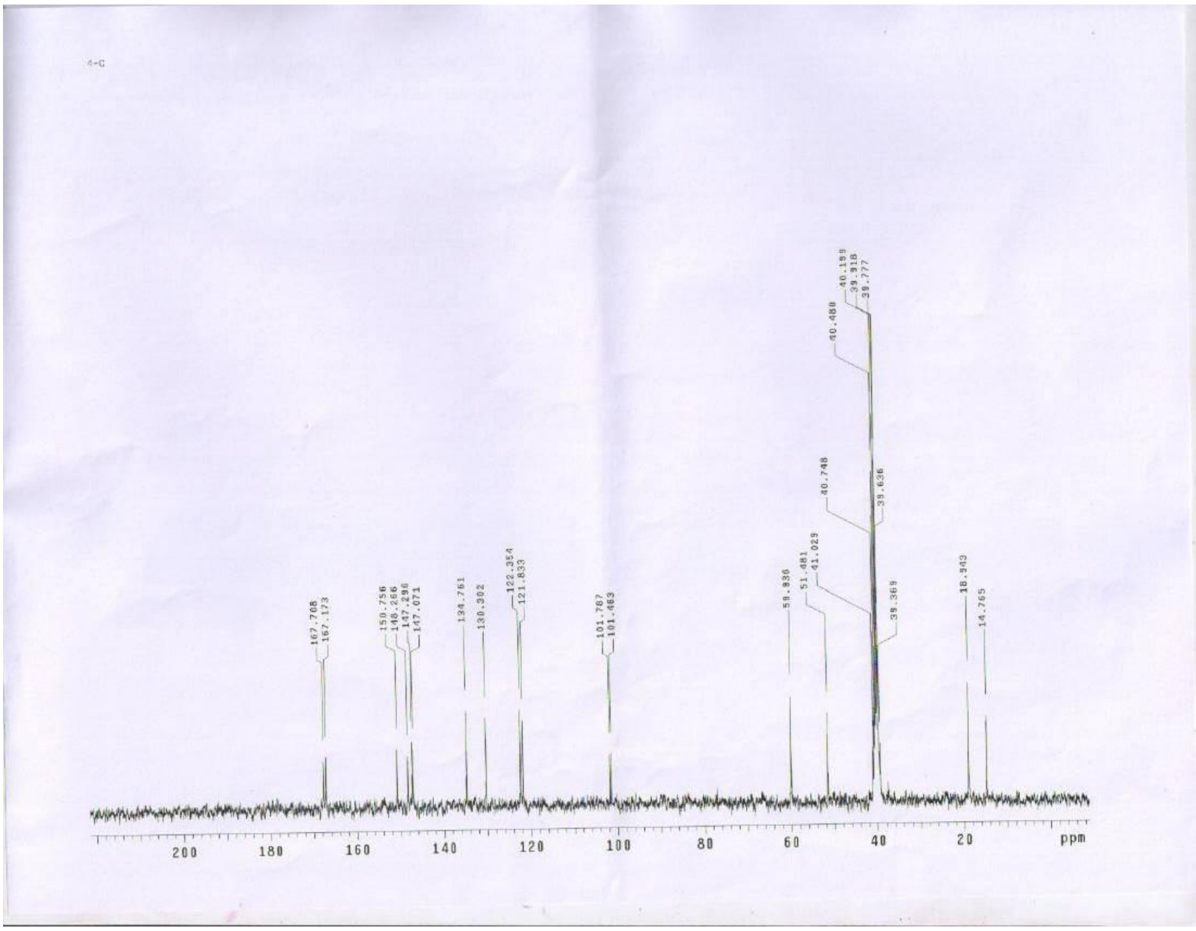

Figure S13. MS spectrogram of Compound **17**.

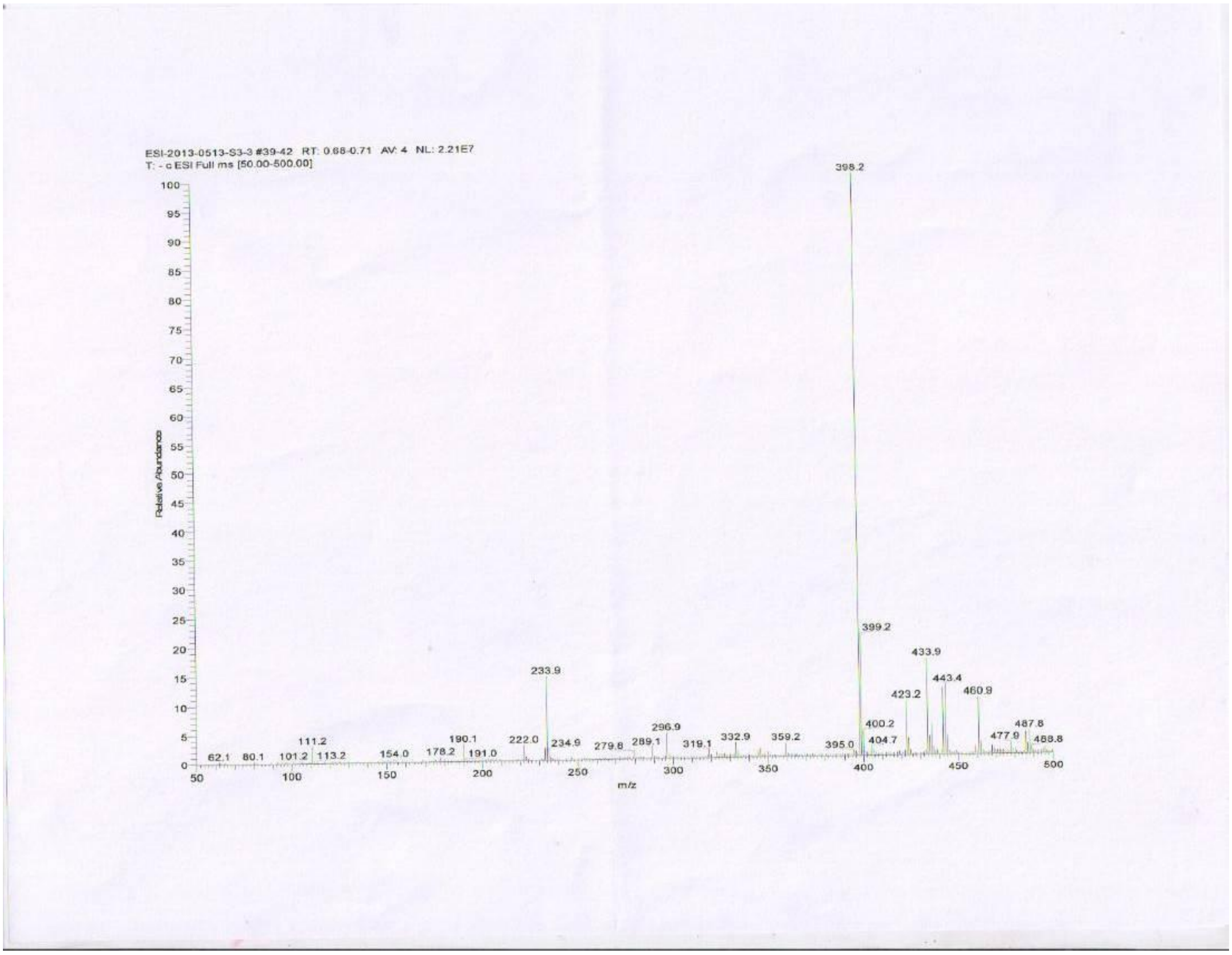

Figure S14. ¹H-NMR spectrogram of Compound **17** in DMSO-d_6.

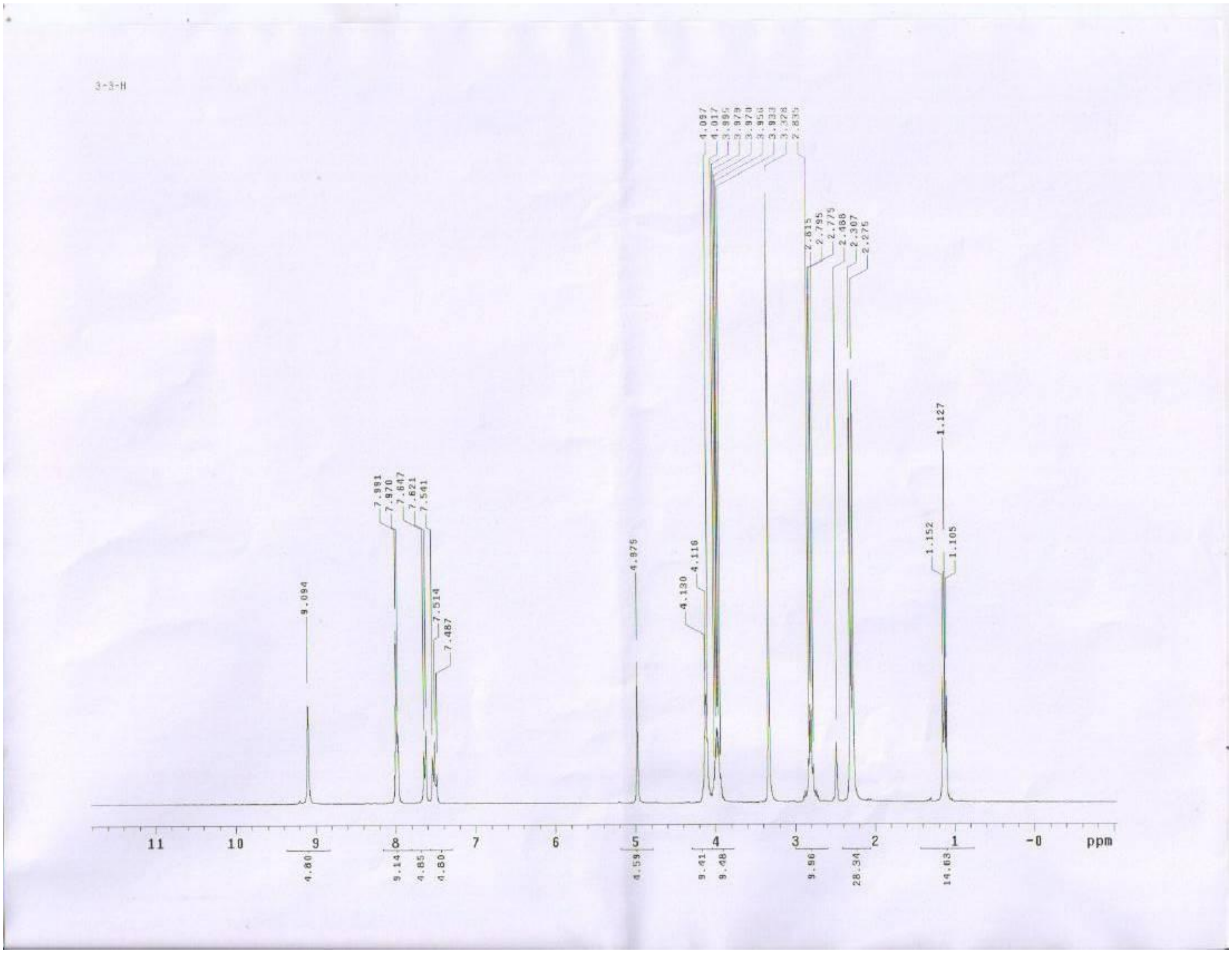

Figure S15. ^{13}C-NMR spectrogram of Compound **17** in DMSO-d_6.

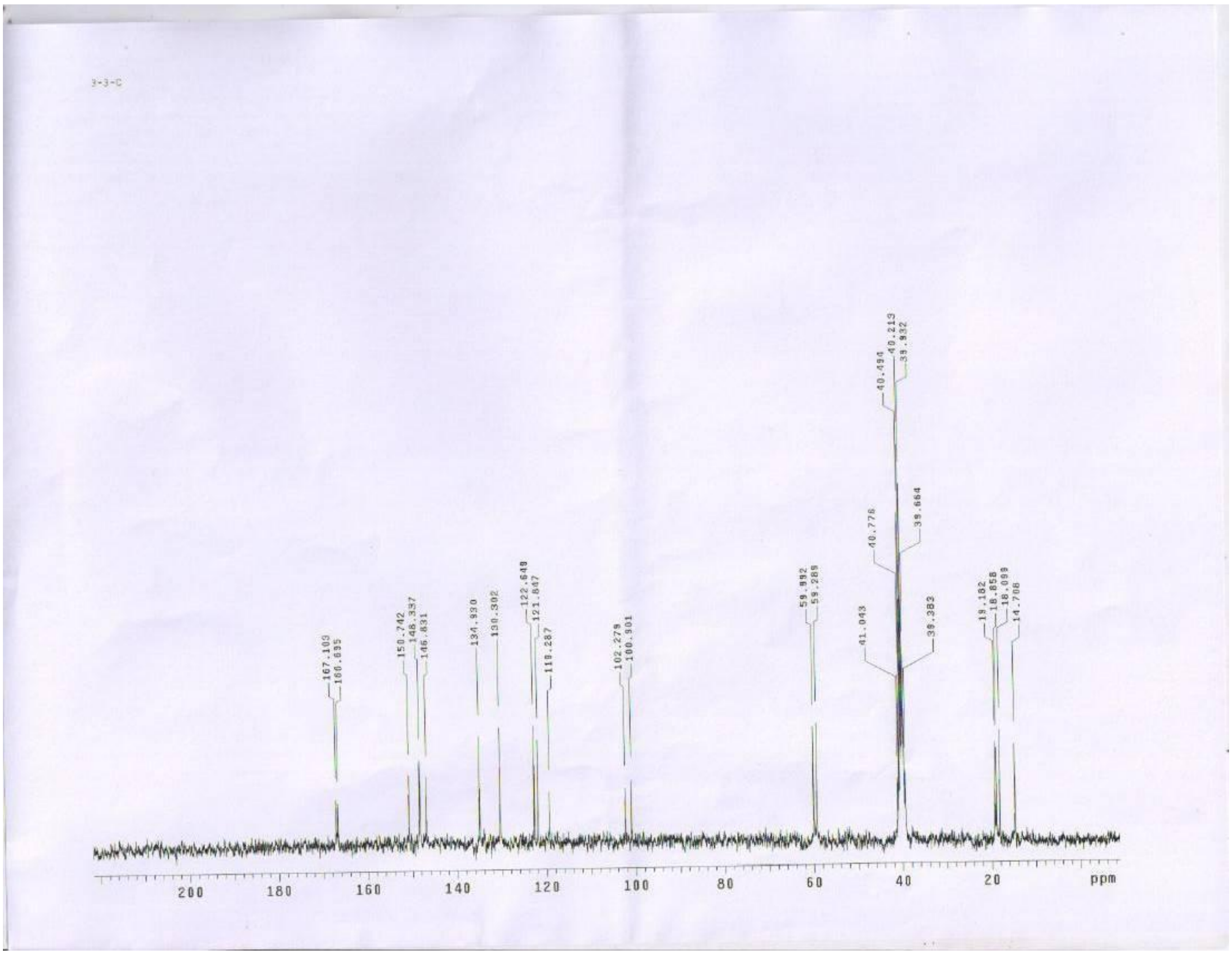

Figure S16. MS spectrogram of Compound **18**.

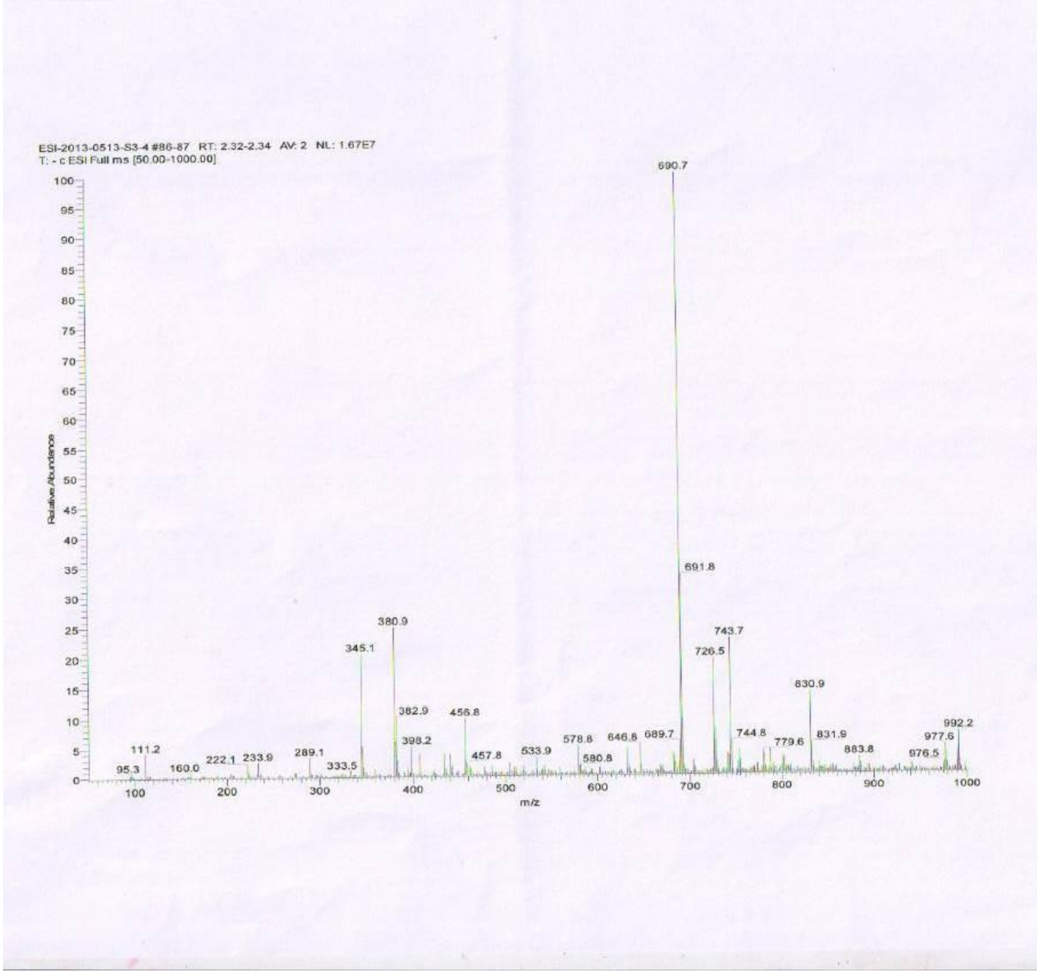

Figure S17. ^{1}H-NMR spectrogram of Compound **18** in DMSO-d_6.

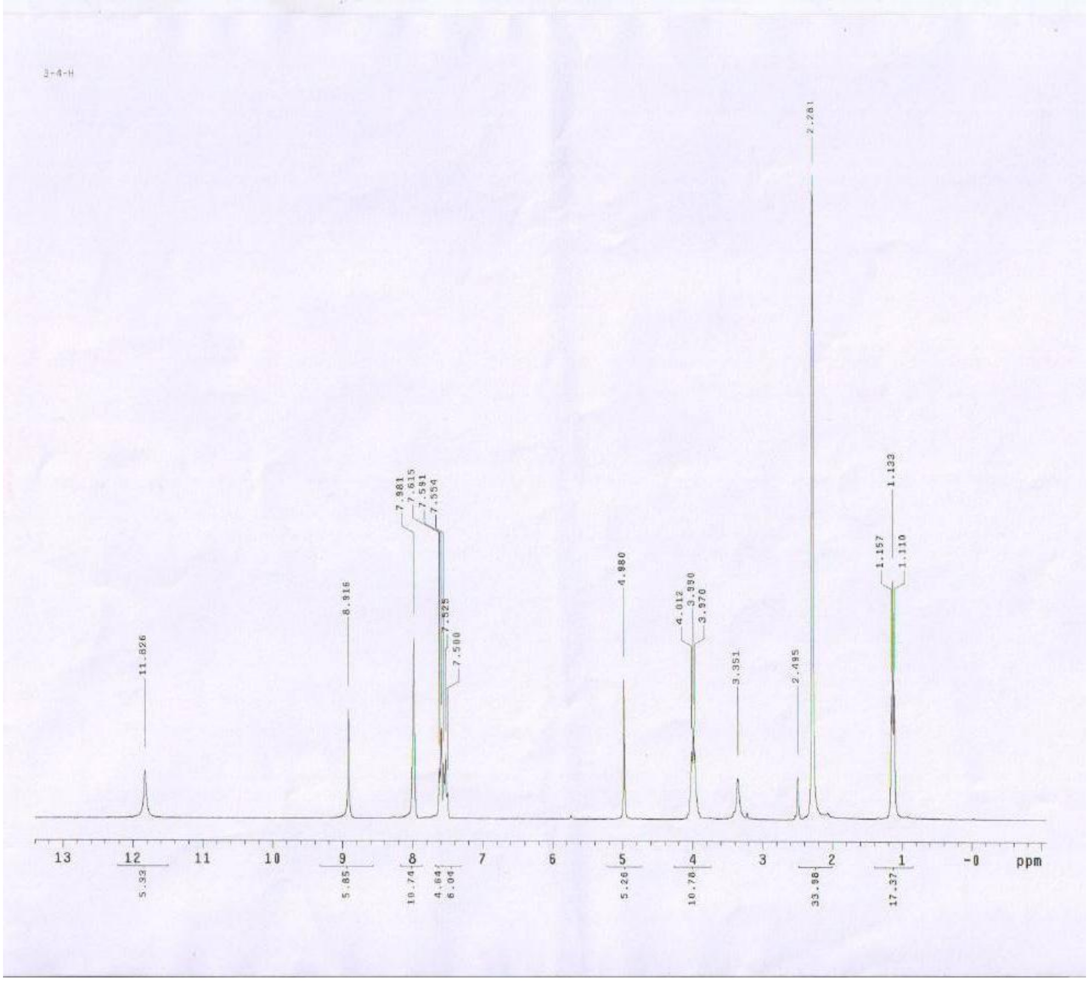

Figure S18. ^{13}C-NMR spectrogram of Compound **18** in DMSO-d_6.

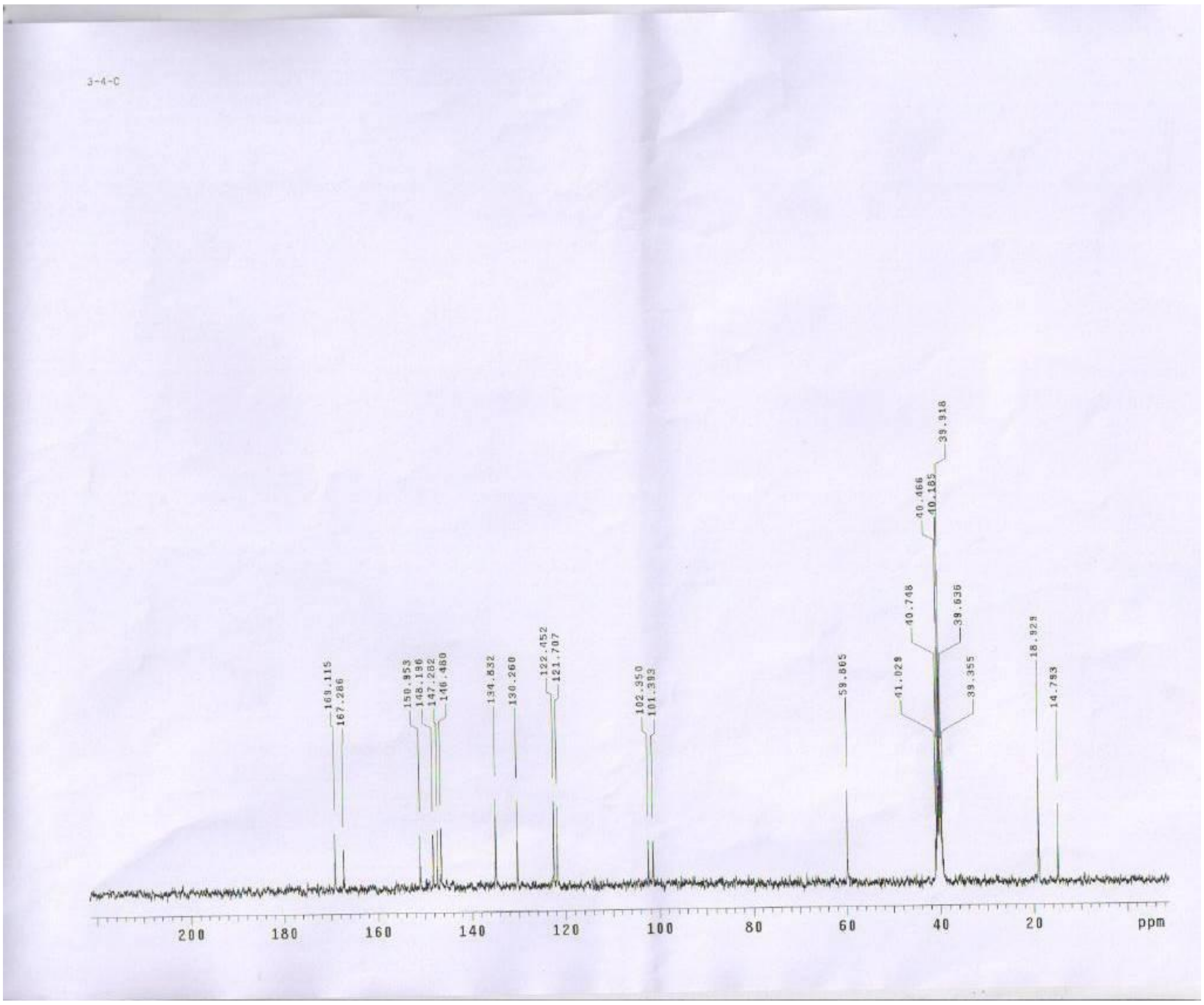

Figure S19. MS spectrogram of Compound **19**.

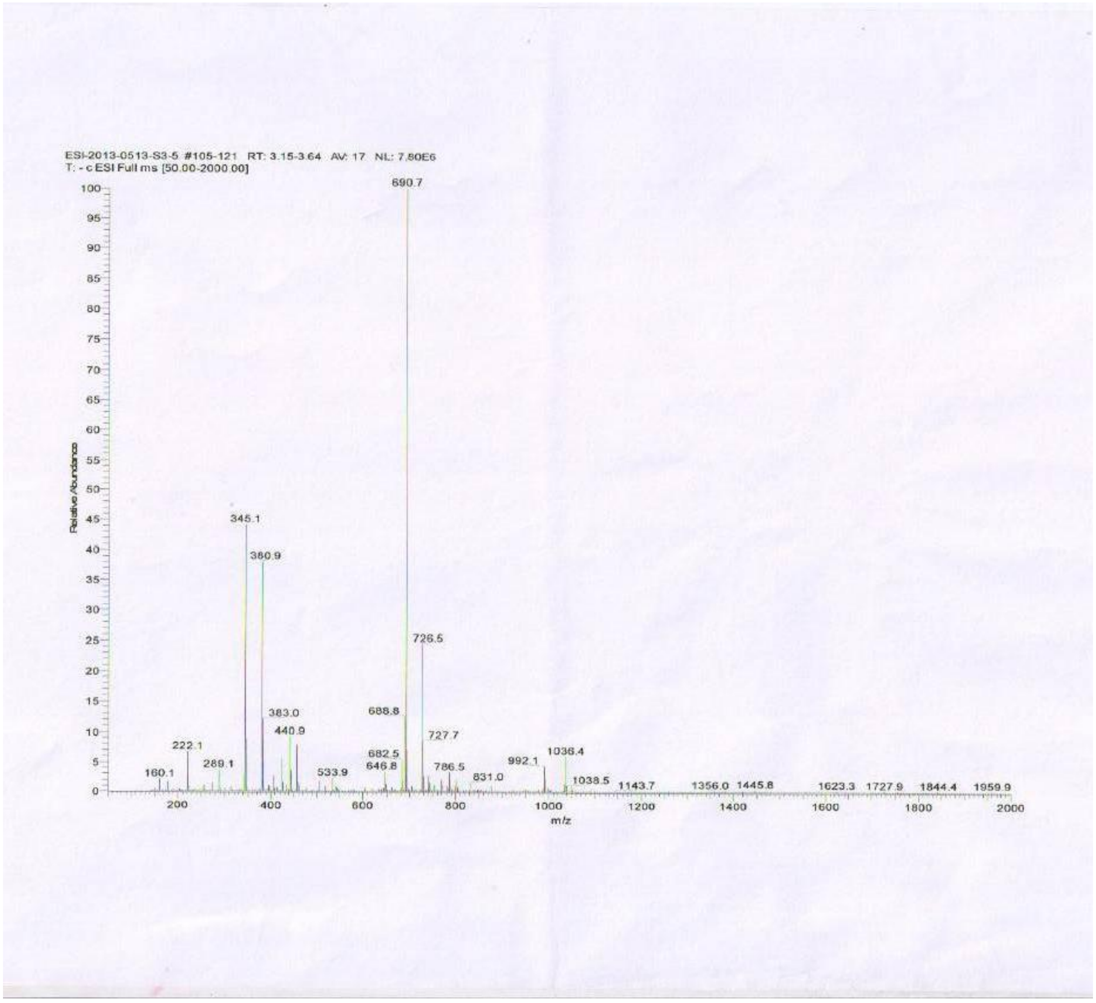

Figure S20. ^{1}H0-NMR spectrogram of Compound **19** in DMSO-d_6.

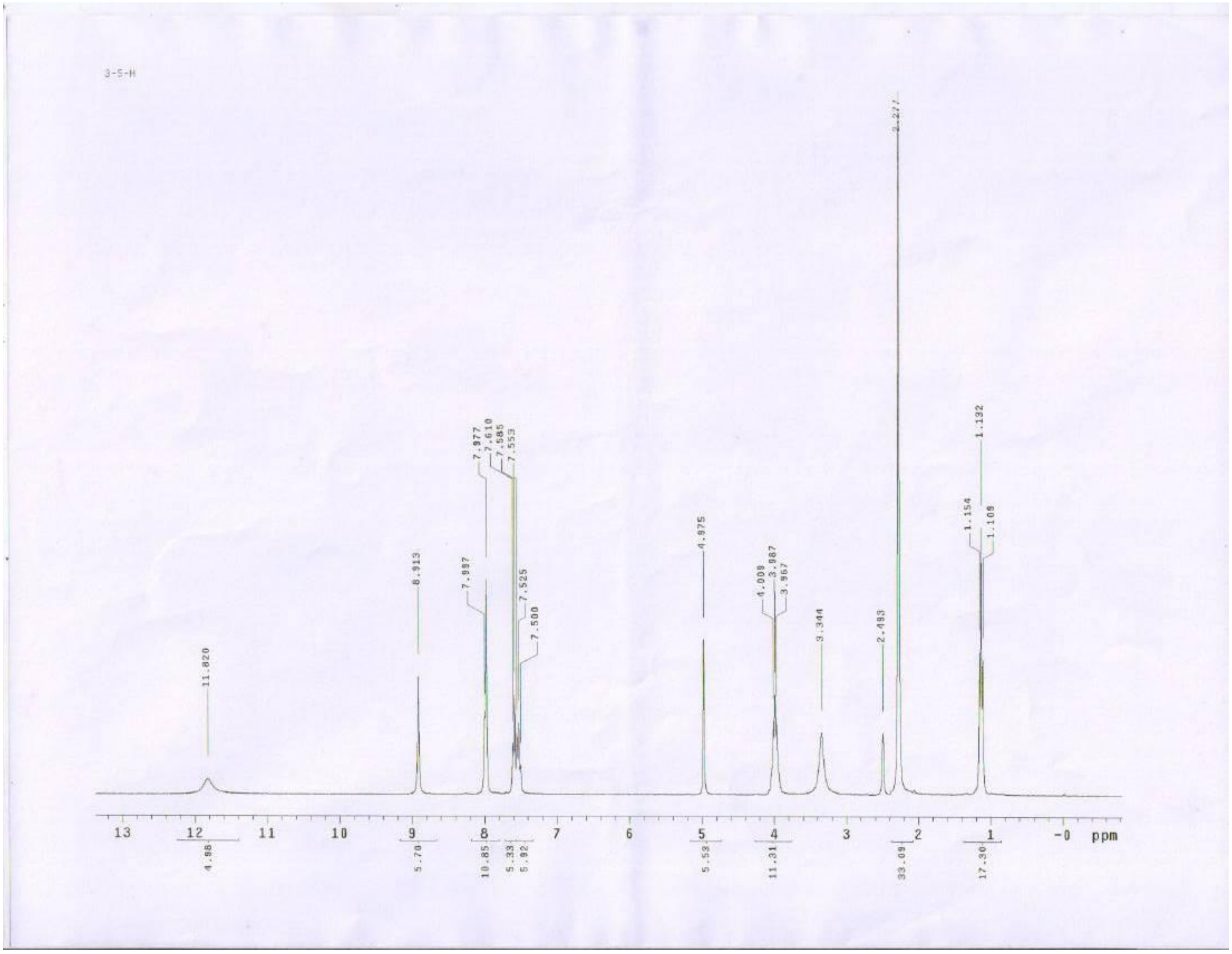

Figure S21. ^{13}C-NMR spectrogram of Compound **19** in DMSO-d_6.

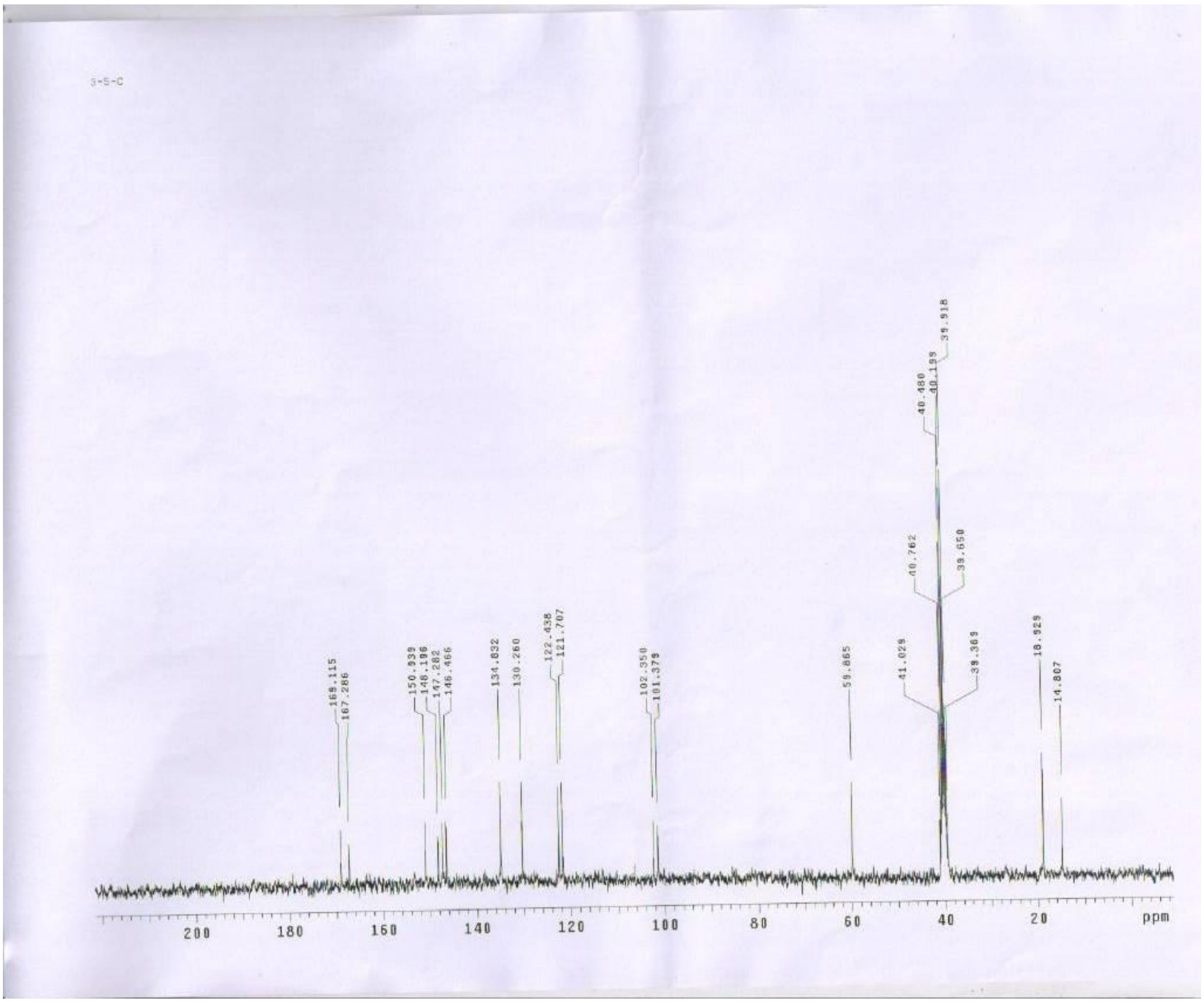

Figure S22. HPLC Chromatogram of Impurity **5**.

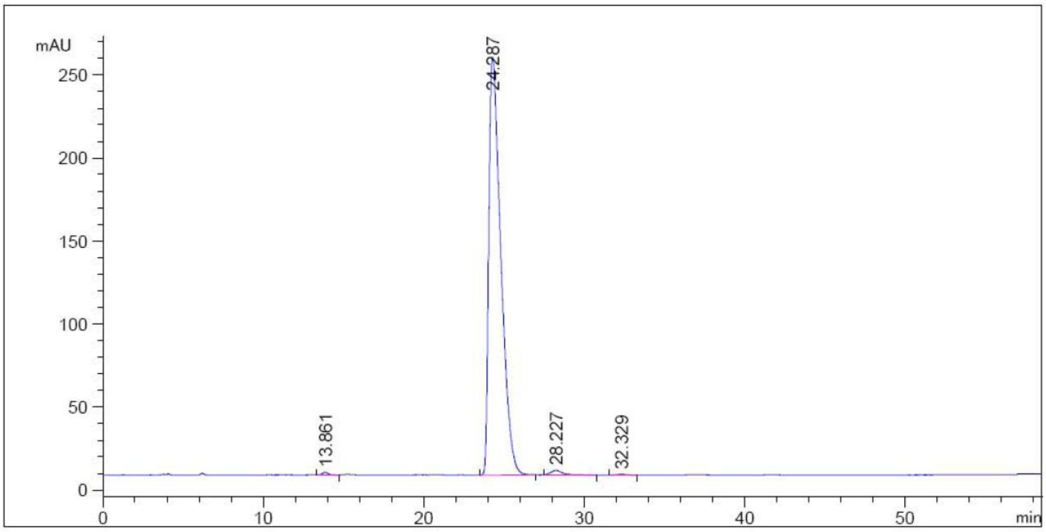

===

Area Percent Report

===

Sorted By : Signal
Multiplier : 1.0000
Dilution : 1.0000
Use Multiplier & Dilution Factor with ISTDs

Signal 1: VWD1 A, Wavelength=238 nm

Peak #	RetTime [min]	Type	Width [min]	Area mAU *s	Height [mAU]	Area %
1	13.861	MM	0.4149	42.41013	1.70344	0.3161
2	24.287	BB	0.7832	1.31751e4	251.99783	98.1915
3	28.227	MM	0.9895	165.30312	2.78439	1.2320
4	32.329	MM	0.8895	34.95171	6.54885e-1	0.2605

Totals : 1.34178e4 257.14055

===

*** End of Report ***

Figure S23. MS spectrogram of Impurity **5**.

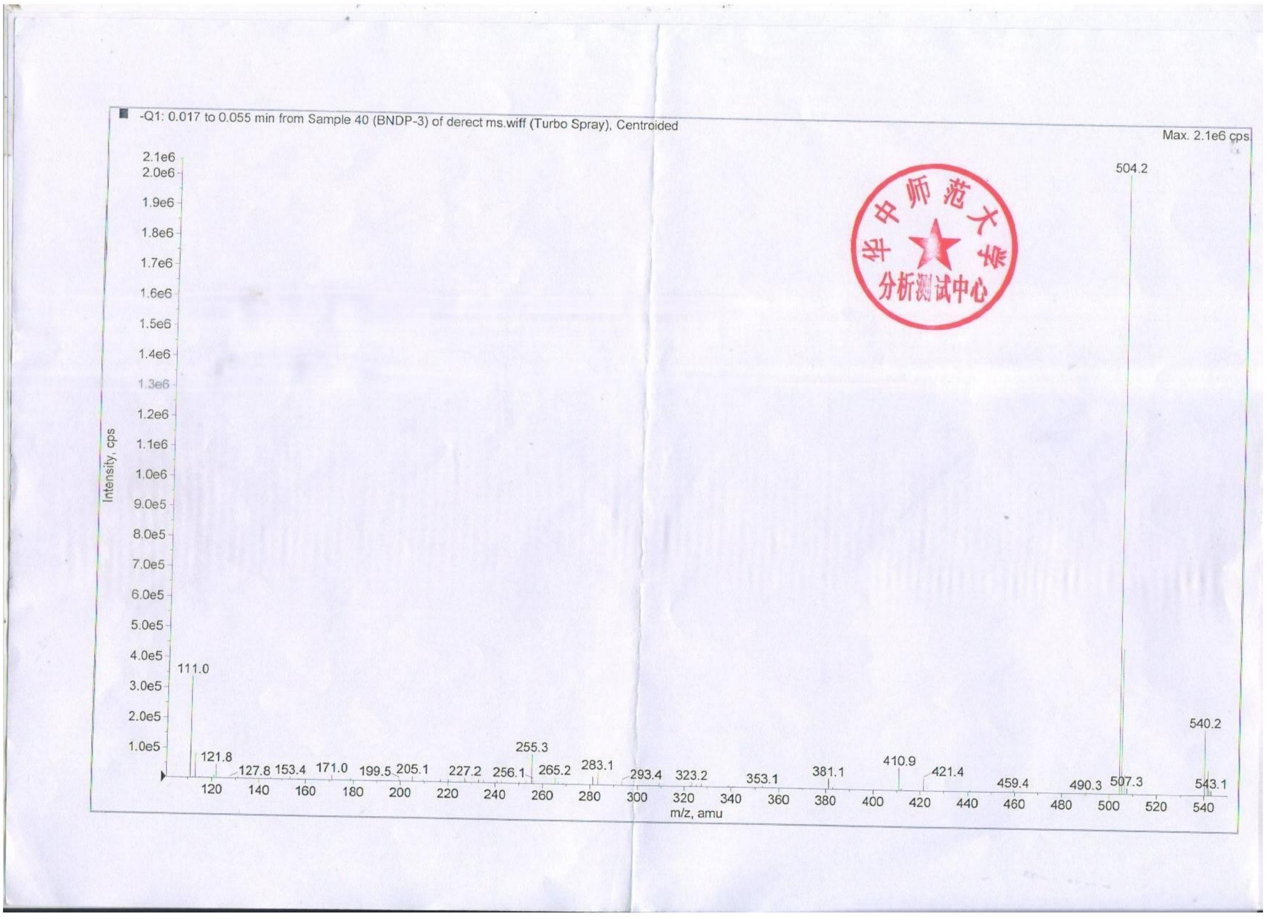

Figure S24. ^{1}H-NMR spectrogram of Impurity **5** in $CDCl_3$.

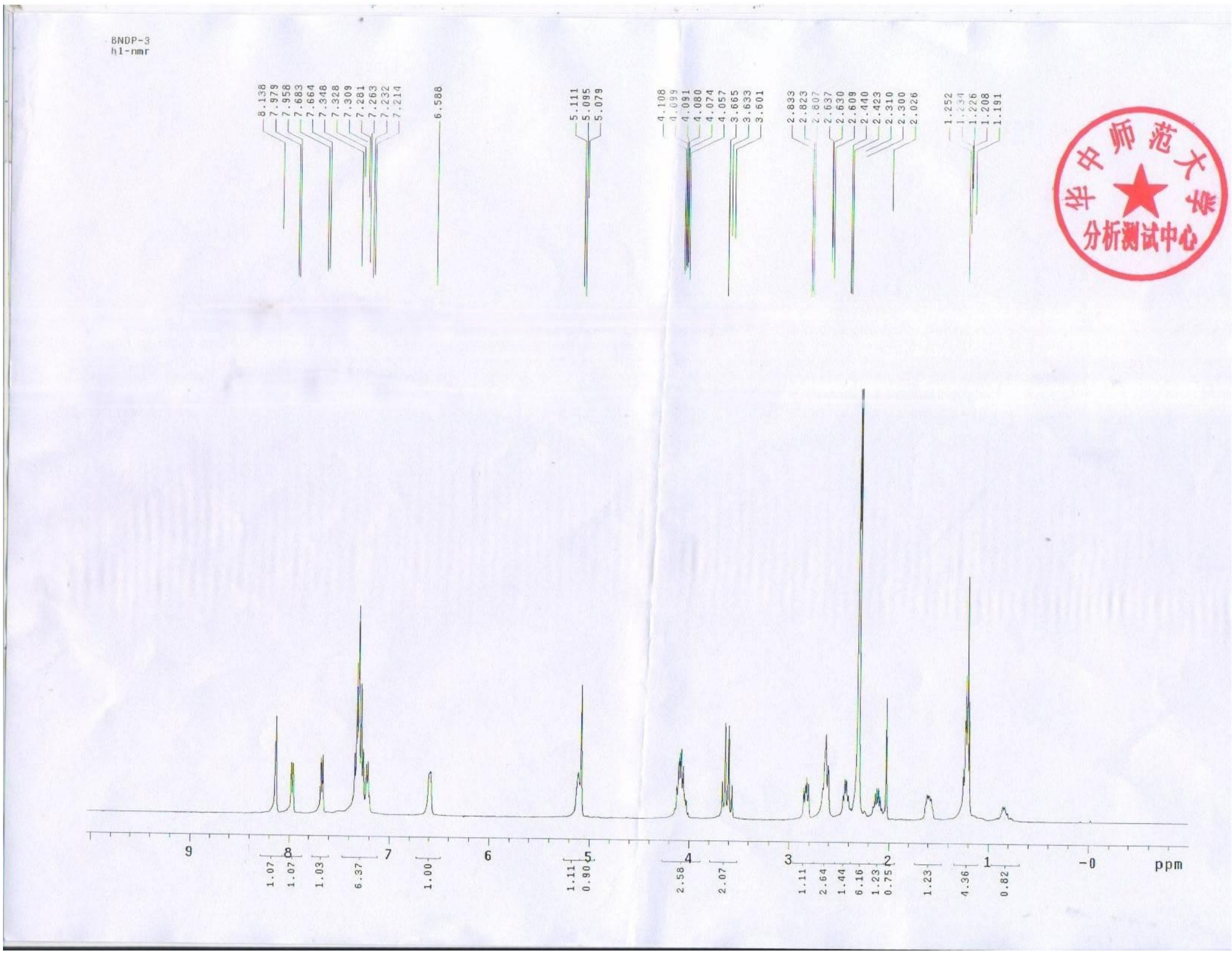

Figure S25. ^{13}C-NMR spectrogram of Impurity **5** in $CDCl_3$.

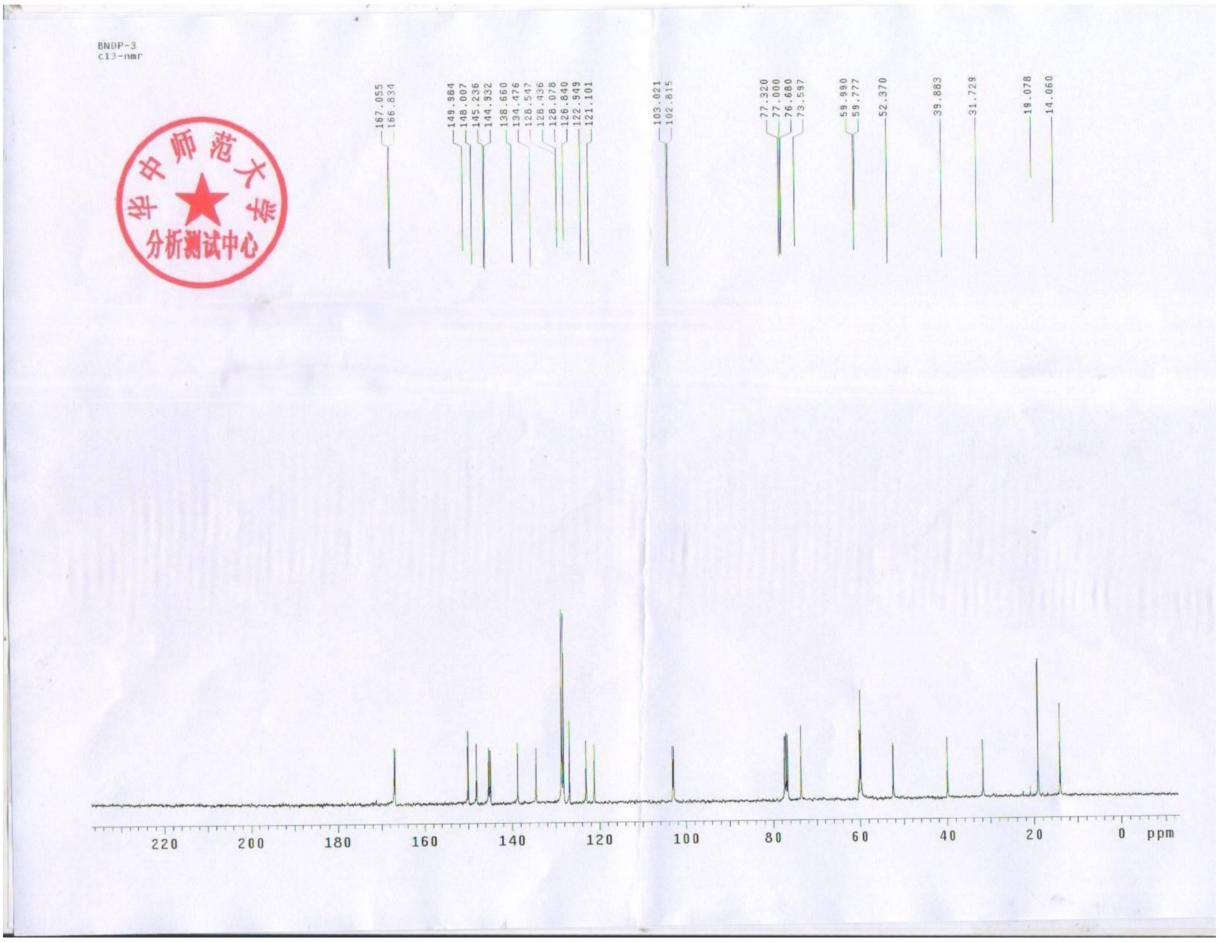

Supplement: Supplementary file 1 [file molecules-19-01344-s001.pdf]
